# Voltage- and pH-driven evolution of multi-pathway C–C coupling in CO_2_ electroreduction on copper

**DOI:** 10.1039/d5sc05367f

**Published:** 2025-10-06

**Authors:** Chengyi Zhang, Ziyun Wang

**Affiliations:** a School of Chemical Sciences, University of Auckland Auckland New Zealand ziyun.wang@auckland.ac.nz

## Abstract

Reducing CO_2_ into high-energy-density multi-carbon products is critical for addressing climate and energy challenges, with copper being the only metal capable of catalyzing this transformation. However, the fundamental mechanisms of C–C coupling on copper surfaces remain elusive. Previous models have primarily focused on *OC–CO and *OC–COH coupling steps, lacking the dynamic and comprehensive perspective towards the whole system. Addressing this gap, our microkinetic modeling systematically investigates how environmental factors modulate multi-pathway C–C coupling mechanisms. We demonstrate that voltage and pH do not simply enhance a single coupling step but dynamically regulate the accessibility and competition among multiple coupling routes, consistent with previous experimental research. These findings establish a more comprehensive understanding of C–C coupling under realistic electrochemical conditions, offering new guidance for the rational design and optimization of copper-based catalysts for sustainable multi-carbon product synthesis.

## Abstract 2

This study reframes CO_2_ electroreduction C–C coupling as an evolving mechanistic framework: a dynamic, multi-pathway process shaped by voltage and pH, overturning intuition and guiding catalyst design under realistic conditions.

## Graphical abstract

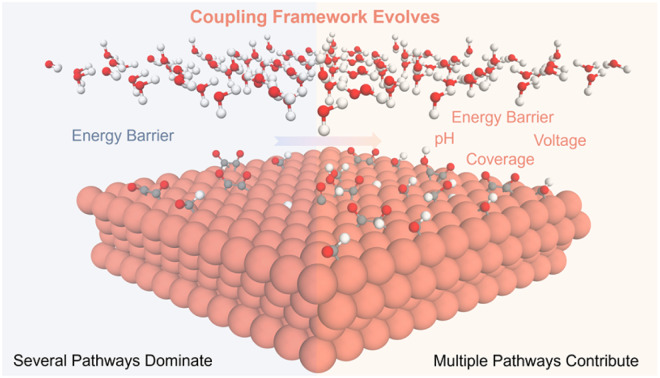

## Introduction

Electrochemical CO_(2)_ reduction reaction (CO_2_RR) has emerged as a cornerstone technology for sustainable energy solutions and a carbon-neutral future.^1–4^ By directly converting CO_2_, a primary greenhouse gas, into valuable chemicals and fuels, CO_2_RR offers the pathway to mitigate climate change. It creates a platform for storing renewable energy, such as solar and wind energy. Among various products generated from CO_2_RR, C_2_ and higher carbon compounds, such as ethylene, ethanol, and acetate, are particularly appealing due to their higher energy density. However, achieving efficient and selective production of C_2_ products remains a formidable challenge. Copper (Cu) is unique among various catalysts because it reduces CO_2_ to produce high hydrocarbon products other than carbon monoxide or formate.^5,6^ However, high overpotential and low selectivity toward the target products remain obstacles for industrial applications of CO_2_RR on Cu-based materials. The heart of producing C_2+_ products is the C–C coupling step, the critical reaction determining the formation of multi-carbon species.^7,8^ This step is essential for designing catalysts with enhanced activity and selectivity towards C_1_ and multi-carbon products.

Recent research efforts have focused on unraveling the mechanisms underlying C–C coupling on Cu surfaces based on the identified intermediates on the Cu surface. Hwang and co-workers employed the time-resolved attenuated total reflection-surface enhanced infrared adsorption spectroscopy (ATR-SEIRAS) to observe the *OCCO intermediates during the CO_2_RR process on electrodeposited Cu.^9^ Koper and co-workers detected the *OCCOH intermediates using the Fourier transform infrared spectroscopy at low overpotential.^10^ Due to the transient nature of intermediates and the difficulty in directly observing atomic behaviours, the coupling mechanism is primarily explored through theoretical research. Kopper and co-workers proposed the *HOC–CO coupling mechanism towards the C_2_H_4_ and EtOH products on the Cu(100) surface.^10^ Xiao *et al.* proposed the *OC–CO dimerization on the oxidized Cu matrix and *OC–COH pathway at neutral pH, with *CO dimerization dominating at high pH on the Cu(111) surface.^11,12^ Cheng *et al.* adopted the full-solvent model to investigate the CO dimerization on the Cu(100) surface.^13^ In their microkinetic modeling, Liu *et al.* proposed incorporating both *OC–CO coupling and *OHC–CHO dimerization pathway.^14^ Montoya *et al.* compare the coupling barrier of CO on Cu(111) and Cu(100) surfaces, indicating that Cu(100) will favor the *OC–CO reaction at low potential.^15^ Carter and co-workers pointed out the better accuracy of the embedded correlated wavefunction (ECW) theory and tested the activation barrier of various coupling mechanisms, incorporating the *HOC–CHO, *HOC–COH, *OC–COH, *OC–CO, *OC–CHO, and *OHC–COH pathways, indicating the three kinetically feasible pathways: *HOC–CHO, *HOC–COH, and *OHC–CHO.^16^ Qiao and co-workers adopted machine learning (ML) strategies to investigate six precursors (*CO, *COH, *CHO, *CH, *CH_2_, and *CH_3_) and 21C_2_ corresponding combinations.^17^

However, these calculations primarily focused on the activation barrier and Gibbs energy difference of different coupling reactions. Moreover, the coupling reaction is the non-proton-coupled electron transfer (non-PCET) step, indicating that the applied voltage may affect this reaction minimally. In contrast, the applied voltage could significantly accelerate the hydrogen evolution reaction (HER). Relying solely on the mechanism where coupling predominantly occurs through one specific reaction (mainly through the *OC-CO reaction with an activation barrier of approximately 0.8 eV)^8^ would lead to the conclusion that the selectivity of the C_2_ product should diminish with the increased applied voltage and never compete with HER. This is because both *CO adsorption and *OC–CO coupling reactions are non-PCET (non-electrochemical) steps, and thus their associated barriers are largely insensitive to electrode potential. In contrast, the rate-determining step (RDS) for HER is commonly understood as the Volmer step (H^+^ + e^−^ → *H), a PCET step. This barrier decreases substantially with increasing overpotential, often reaching ∼0.8 eV at ∼0 V *vs.* RHE and becoming even lower at more negative potentials.^18^ Therefore, under such a framework, where HER is PCET-driven and C–C coupling occurs only *via* *OC–CO coupling, one would expect the faradaic efficiency (FE) of H_2_ to increase monotonically with applied potential, while the FE of C_2_ products should correspondingly decrease.^5^

However, this prediction contradicts experimental observations, which show that C_2_ selectivity initially increases with more negative potentials, surpassing H_2_ production at moderate overpotentials, before eventually declining. This suggests that C–C coupling is not governed solely by *OC–CO dimerization, and that other coupling pathways—particularly those involving hydrogenated species may play a significant role in enhancing C_2_ formation at intermediate potentials. While experimentally observed intermediates exist, the proposed mechanisms have successfully guided experiments. This suggests that some critical factors may have been overlooked. In this study, we aim to investigate the mechanism of C–C coupling by systematically exploring the hydrogenation pathways of possible C_1_ intermediates (including *CO, *COH, *CHO, *CHOH, *CH, and *CH_2_).^11^ We then exhaustively examined 21 potential coupling mechanisms based on the reaction energetics estimated by explicit-solvent calculations. Due to the extensive reaction pathways following coupling and our primary focus on understanding how coupling mechanisms vary under different voltages and pH levels, we assumed that the C_2_ intermediates formed after the coupling step to the final products C_2_H_4_ are downhill in energy space, consistent with previous research.^14^ Our results align well with prior experimental data.^6^ First, we analyzed the coupling barriers for various intermediates and observed that the coupling barrier decreases progressively as the hydrogenation of C_1_ intermediates. Next, we performed microkinetic calculations based on these barriers. The microkinetic results showed excellent agreement with experimental trends, revealing that the selectivity towards C_2_ product formation initially increases with applied voltage, intersects with HER, reaches a peak, and decreases during the FE of the methane increase at high applied voltage, showing the similar trend to the previous microkinetic study.^19–21^ Further analysis under pH 13 highlighted the intermediates and the fastest coupling reactions at different voltages, with surface coverage results consistent with experimental characterizations. Finally, based on the microkinetic model, we identified the most favorable coupling pathways across varying pH levels and voltages. This study provides a comprehensive framework for understanding the evolution of coupling mechanisms under diverse electrochemical conditions, providing valuable insights for advancing catalyst design and enhancing the selectivity of C_2_ products in CO_2_RR.

## Results and discussion

**Fig. 1:**
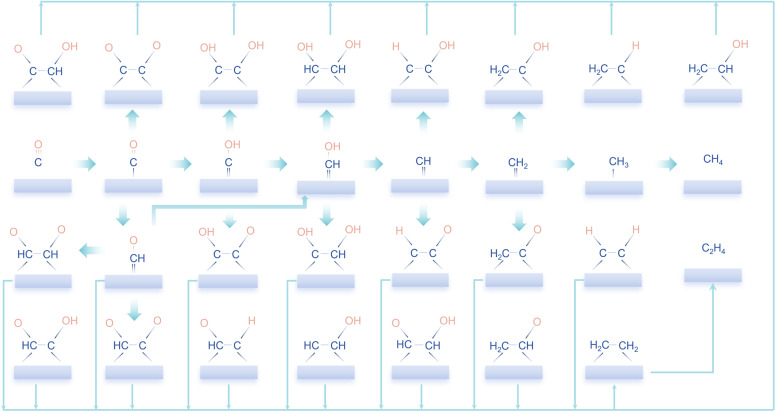
The sketch of the reaction pathway in this work involves six possible C_1_ precursors and all 21 corresponding combinations. To make the image look neater, we have omitted some arrows for coupling reactions.

Since CO is a key intermediate for coupling reactions in CO_2_RR, we systematically simulate all possible coupling pathways by starting with CO gas and progressively hydrogenating it to methane, following Goddard's work.^11^ Both Eley-Rideal (ER) and Langmuir–Hinshelwood (LH) mechanisms were directly considered in our microkinetic modelling. Since *CH_2_OH and *CH_3_ are saturated intermediates with relatively stable chemical properties, their reactivity on Cu(111) is significantly reduced. Therefore, six key intermediates were identified as potential precursors: *CO, *COH, *CHO, *CHOH, *CH, and *CH_2_. The various combinations of these intermediates yield 21 distinct coupling products, as shown in Fig. 1.

Considering the vast number of possible pathways involving C_2_ intermediates, it becomes impractical to evaluate each subsequent reaction in detail. Our primary focus lies on the coupling reaction, which is critical for the overall selectivity and efficiency of C_2_ products. Notably, many coupling mechanisms in our mechanism involve intermediates that lack oxygen. To streamline the study while reflecting the complete CO_2_RR process as accurately as possible, we consolidated all the C_2_ intermediates after coupling them into the most representative and experimentally dominant product, ethylene (C_2_H_4_).^6^ Ethylene is widely accepted as the primary product of C_2_ intermediates on Cu in CO_2_RR, making it the natural choice for simplification. Furthermore, based on previous research, we assume that the energy landscape from C_2_ intermediates to ethylene is involved in the energetic downhill process.^14,22^ This assumption allows us to focus exclusively on the coupling step without overcomplicating the number of branching pathways that occur post-coupling.

### CC coupling barrier

**Fig. 2:**
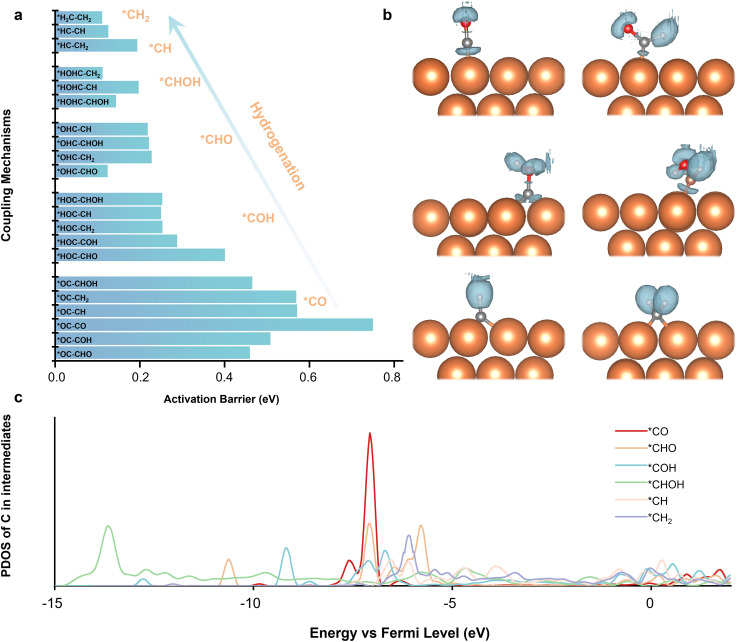
(a) The activation barrier of the 21 coupling mechanisms. (b) The ELF of C_1_ precursors. (c) The PDOS of C in C_1_ precursors.

Fig. 2a demonstrates a clear trend where the activation barriers decrease progressively as the C_1_ precursor transitions from *CO to *COH, or *CHO, then *CHOH, and finally to *CH and *CH_2_. This systematic reduction in the coupling barrier suggests an inherent relationship between the intrinsic bonding characteristics of the intermediates and their coupling behavior. To explore the underly reason, we conducted the electron localization function (ELF) and partial density of states (PDOS) for each intermediate. From the perspective of the transition state, it involves the breaking of the original bond and the formation of a new bond. The nature of the C–C coupling process can be understood as breaking an existing bond on the carbon atom and forming a new C–C bond. The strength of the original bond directly influences the coupling barrier: the stronger the bond, the more energy is required to break the original bond. Therefore, analyzing the bonding characteristics of each C_1_ intermediate is crucial to explain the observed trend.

As presented in Fig. 2b, ELF analysis reveals a particular amount of localization of electrons in the C–O bond in *CO intermediates. This indicates a strong interaction between the carbon and oxygen atoms, contributing to the relatively high coupling barrier of *CO. However, as the intermediates undergo successive hydrogenation, the ELF around the carbon atom decreases from the *CO to *COH, *CHO, then *CHOH. When the intermediates reach the *CH and *CH_2_, the localization electron is predominantly distributed on the hydrogen atoms, signifying the weakening of the original C bonds. This redistribution of the electron makes it easier for the carbon atom to break its original bond and form a new C–C bond, thereby lowering the coupling barrier.

Consistent with the ELF analysis, PDOS calculations further support these findings; we analyze the PDOS of the carbon atoms of each intermediate, as presented in Fig. 2c. The results further support these findings. The PDOS of *CO exhibits a sharp and prominent peak, indicating a highly localized electronic state and a strong bond character. As hydrogenation proceeds, the PDOS peaks become increasingly delocalized and diminish in intensity, signifying a gradual reduction in bond strength and an increasing ease of bond dissociation. The DOS peaks are significantly reduced when the intermediates reach *CH and *CH_2_. Aligning with the lower coupling barriers observed in these species. These findings highlight a clear trend: the coupling process becomes more favorable as the hydrogenation process progresses. The reduction in coupling barriers can be attributed to the weakening of the original bonds on the carbon atom and the increasing delocalization of the electronic states. We also calculated the potential-dependent energy barriers for different coupling mechanisms on the Cu(100) surface and found trends consistent with those observed on Cu(111). It indicates that the modelling on the Cu(100) model for the investigation of the multi-coupling reaction network will present a similar trend to that on Cu(111). Besides, as Cu(111) is the most stable low-index facet of copper, it provides a well-defined surface that facilitates mechanistic clarity and reproducibility.^16,23,24^ To maintain a clear focus on the mechanistic insights of the coupling processes, we selected Cu(111) as the representative surface for in-depth analysis in this study.

### CORR at pH 13

After analyzing the energy-space coupling mechanism, we conducted comprehensive microkinetic modeling based on the calculated energy barriers. Microkinetic modeling is an essential tool for studying complex catalytic reactions. It integrates complicated reaction networks and the influence of environmental factors like pH, applied voltage, and partial pressure. By constructing the reaction network under different conditions, we can gain insights into the reaction rates, selectivity, and dominant pathways.

**Fig. 3:**
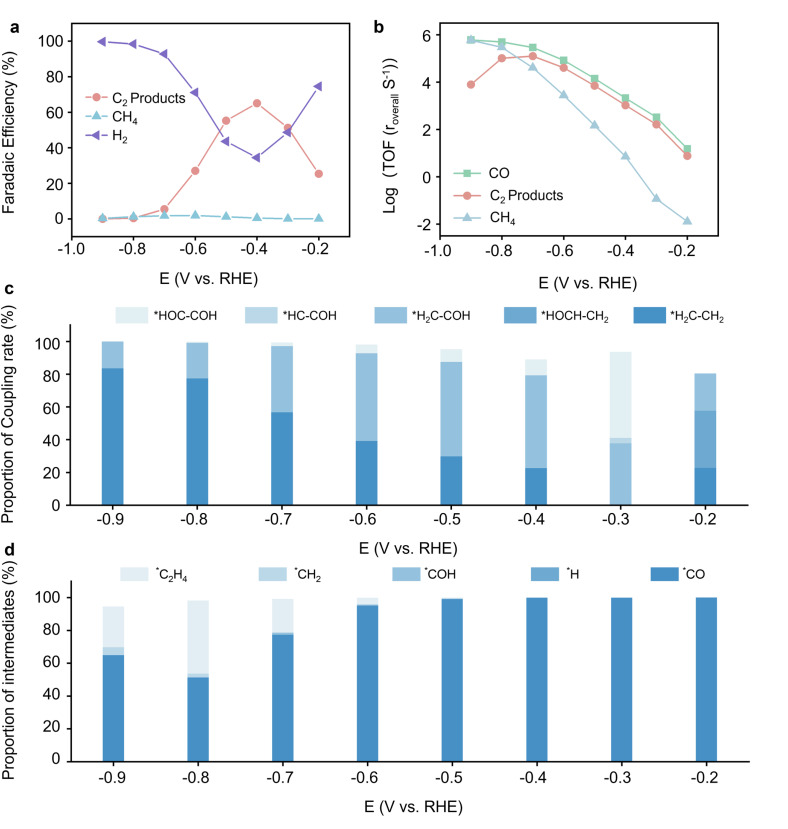
Microkinetic modeling results under different voltages at a pH of 13. (a) The selectivity towards H_2_, CH_4_, and C_2_ products. (b) The turnover frequency (TOF) of CO, CH_4_, and C_2_ products. TOF of CO indicates the consumption rate of CO. (c) The proportion of the top three coupling rates to total coupling rates under different voltages. (d) Proportions of different intermediates to total intermediate coverage under different voltages.

Our results agree with previous experimental findings, confirming the validity of our model, as presented in Fig. S31 and S32. When considering the selectivity of the reaction, as illustrated in Fig. 3a, we observed that the selectivity of the C_2_ products initially increases with the applied voltage, reaching a maximum, and then decreasing. In contrast, the selectivity of the HER decreases initially and then increases at higher voltages. Within a moderate voltage range, the C_2_ products and HER selectivity intersect. Such trends demonstrate the delicate balance between reaction pathways under different electrochemical conditions. For HER, the RDS is the Volmer step, which involves hydrogen adsorption from water onto the catalyst surface with an initial activation barrier of about 0.8 eV and decreases linearly with increasing applied voltage. Since this step is a PCET process, the HER rate increases exponentially with the applied voltage within the reaction potential range. On the other hand, both the CO adsorption step and the *OC–CO coupling step in CORR are chemical steps. If the C_2_ products proceed entirely through the *OC–CO coupling step, the highest barrier for this pathway is approximately 0.8 eV. Under such conditions, the selectivity of C_2_ products cannot surpass that of HER. This finding suggests that alternative coupling mechanisms must dominate the C_2_ product formation. Besides, if only a single coupling mechanism were adequate, the reaction activity and selectivity of C_2_ products would be expected to exhibit a nearly linear decline beyond a specific voltage when the activation barrier of the hydrogenation is lower than the coupling of the precursor. However, both previous experimental findings and our simulation results show that this decline is significantly moderated, appearing much smoother than anticipated. This observation suggests that, in the CORR and CO_2_RR processes, C–C coupling is not dominated by a single mechanism. Instead, it likely involves multiple coupling pathways that dynamically shift and coexist under different voltage conditions. This dynamic interplay between various coupling mechanisms ensures that the activity and selectivity of C_2_ products remain more stable across a broader voltage range, leading to the observed smoother trend. To further validate the consistency between our microkinetic modeling and experimental observations, we performed a Tafel slope comparison between our modeling results and Hori's experimental data, as shown in Fig. S37. Both the experimental measurements and the modeling results confirm that the formation rates of C_2_ products are relatively insensitive to potential compared to C_1_ and H_2_ changes in SHE scale. This consistency further supports the reliability of our coupling kinetic analysis. Fig. 3b further reveals the activity trends of CO, C_1_ products, and C_2_ products under varying voltages. At lower voltages, C_2_ products dominate the reaction. However, when the applied voltage exceeds approximately −0.7 V *vs.* RHE, the activity of C_2_ products begins to decline, and the C_1_ products gradually surpass the C_2_ products in selectivity and activity. This trend aligns closely with previous experimental studies, reinforcing the reliability of our model. Notably, with the further increase of the applied voltage, the TOF of the CH_4_ is gradually comparable to CO. This explains why the TOF of the C_2_ products decreases beyond −0.7 V *vs.* RHE. Such a TOF decrease could be attributed to the hydrogenation of CO to CH_4_ involving a set of electrochemical steps, where each hydrogenation step through the ER mechanism experiences a barrier that decreases with increasing voltage until it approaches zero. In contrast, the C–C coupling step is a chemical step. Its activation barrier remains unaffected by the applied voltage. As a result, the coupling step becomes the RDS for the C_2_ product formation at higher voltages. This bottleneck limits the formation of C_2_ products, allowing C_1_ products to dominate the reaction pathway at high applied voltage. Fig. 3c and d presents critical insights into intermediates' coupling mechanisms and coverage behaviors in the electrochemical reduction of CORR under alkaline conditions at pH 13. Fig. 3c indicates that at lower applied voltage, coupling mechanisms involving oxygenated intermediates, such as *COH and *CHOH, dominate. At higher voltages, the proportion of hydrocarbon-based intermediates, like *CH and *CH_2_, coupling increases significantly. This trend is consistent with the PCET process that governs the hydrogenation steps. At low voltages, the hydrogenation of *CO proceeds exceptionally slowly, leading to extremely low concentrations of all following intermediates. Many coupling mechanisms currently play a role simultaneously. However, the overall reaction rates remain very slow. As the voltage increases, the hydrogenation of *CO accelerates, resulting in the intermediate predominantly in the form of carbon–hydrogen–oxygen species such as *CHO, *COH, and *CHOH. Due to the relatively low activation barrier and fast coupling rates of these intermediates, the RDS of the C_2_ products is the hydrogenation of the *CO.

Once these carbon–hydrogen–oxygen intermediates are formed, they are rapidly consumed, making it challenging to observe them in experiments. As the voltage further increases, the conversion rate of *CO to hydrocarbon intermediates like *CH and *CH_2_ increases rapidly. The coupling precursor shifting from oxygenated intermediates to hydrocarbon intermediates is further enabled by the lower coupling barriers of intermediates like *CH and *CH_2_, rendering these species more reactive and dominant at higher voltages. Thus, at higher voltages, the dominance of hydrocarbon intermediates leads to less oxygenated C_2_ products. This dynamic shift aligns with previous literature, indicating that oxygenated C_2_ products like ethanol and acetate are more prevalent at lower voltages, as the kinetic factors favor their formation.^25^Fig. 3d provides another layer of understanding of the coverage behavior. While *CO dominant surface coverages and some intermediates like *OC–CO are frequently detected in spectroscopic studies, they do not play a prominent role in the fastest reactions due to their high hydrogenation and coupling barriers. Instead, hydrogenated products like *COH, *CHOH, *CH, and *CH_2_ dominate the coupling reactions but are challenging to detect due to their rapid consumption. This highlights the importance of combining experimental observations with advanced computational modeling to capture the transient behavior of these intermediates and predict the reaction pathways. The findings also underscore the limitations of solely relying on the surface coverage data to obtain reaction mechanisms, as the most abundant species on the catalysts' surface may not directly participate in the key rate-limiting steps or coupling reactions.

### Coupling mechanisms and coverage under different pH and voltage

**Fig. 4:**
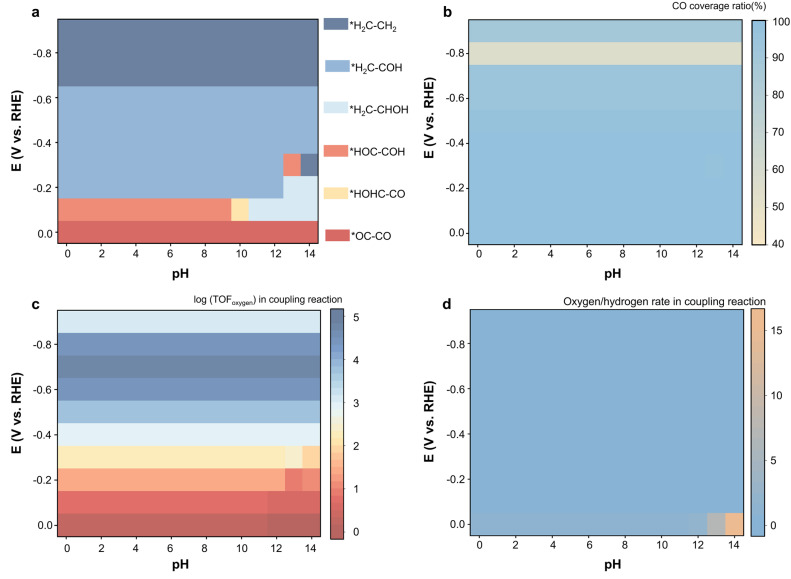
(a) The top coupling reaction mechanism. (b) The coverage of *CO to total intermediates under different pH and voltage. (c) The oxygen-containing precursor coupling rate. (d) The oxygen to hydrogen rate in the coupling reaction.

To expand our conclusion and provide further insights, we systematically explored the electrocatalytic behavior across various voltages ranging from 0.0 to −0.9 *vs.* RHE across a wide range of pH conditions (0–14) in our microkinetic model. We conducted a detailed investigation of reaction mechanisms under different working conditions. At a particularly low applied voltage (−0.0 to −0.2 V *vs.* RHE), the hydrogenation of CO is hindered due to a significant reaction barrier. Consequently, all coupling reactions play a role, and the top three do not dominate the total response. At such low voltage, the hydrogenation of *CO is particularly challenging. Currently, the *CO coverage on the surface is dominant, as illustrated in Fig. S16–S30. The *OC–CO coupling mechanism plays a considerable role in the C_2_ product generation in this regime, regardless of pH, as shown in Fig. S1–S15. As the applied voltage increases, the *OC–CO coupling rate becomes negligible at lower pH, while contributing much at higher pH. This is because the concentration of hydrogen ions increases at lower pH values, accelerating the hydrogenation of *CO compared to high pH levels. As the voltage rises, the *OC–CO coupling mechanism quickly disappears at all pH levels. However, the *CO coverage still dominates; the coupling between *CO and other intermediates, like *CHOH, plays a significant role currently. With further increases in the applied voltage, *CO hydrogenation intensifies. As previously demonstrated, this hydrogenation process reduces the activation barrier, and the coupling between the carbon–hydrogen and carbon–oxygen–hydrogen intermediates is thus preferred. For example, coupling reactions involving *CHOH, *CH, and *CH_2_ gradually dominate. Similarly, the decrease in pH accelerates this transition. As the applied voltage increases, the hydrogenation of *CO becomes severe. The rate of converting *CO to CH_4_ was greatly enhanced. The favor of intermediates proceeds in the reaction pathways. As a result, the *CH_2_ generation is favored at higher voltage, and the *H_2_C–CH_2_ coupling mechanism becomes dominant as its activation barrier is low. This also explains why the FE of oxygenated C_2_ products is much lower in previous experiments. We also consider the coverage effect. Initially, *CO coverage dominates as hydrogenation is challenging. As the applied voltage increases, the hydrogenation process intensifies. However, at this stage, hydrogenation of *CO remains the RDS, and the produced carbon–hydrogenation–oxygen or carbon–hydrogen intermediates quickly react and desorb from the surface, making the coverage of these intermediates extremely low and hard to observe in experiments. Interestingly, the *CO coverage decreases and increases with the applied voltage increase, as shown in Fig. 4b. This is because the desorption of the C_2_ product is the chemical step unaffected by the applied voltage. As the voltage increases, the rate of C_2_ product formation increases, while the rate of the desorption step is not enhanced, making the coverage of the C_2_ products increase. However, regarding the −0.9 V *vs.* RHE, *CO becomes more likely to generate the C_1_ products as each hydrogenation step is the PCET step accelerated with applied voltage increases. Therefore, at higher voltages, the rate of C_2_ product formation sharply decreases, and the CO coverage again dominates the coupling reactions. To simplify our results and give comprehensive guidance, we summarized the fastest coupling mechanisms at different pH values and voltages, as presented in Fig. 4a. This finding aligns well with our previous discussions. We also conducted investigations on the surface coverage of Cu(111) during the CORR under different pH and voltages. The *CO is the predominant intermediate on the surface under most conditions. Except at higher voltage, when the production rate of *C_2_H_4_ increases significantly, the desorption is challenging. However, in all other cases, CO remains the dominant intermediate, maintaining a high surface coverage.

When examining the coupling reaction rates, as shown in Fig. 4a, we found that the type of surface coverage does not directly dictate the coupling mechanism. This indicates that while surface coverage influences the intermediates' distribution, other factors, such as the activation barrier and the reaction rate, are also crucial in determining the reaction pathways. These findings provide valuable insights into both experimental and computational studies, suggesting that having a comprehensive understanding of intermediates, surface coverage, and reaction conditions, rather than any single factor, is key to optimizing CO_2_RR.

### Guidance towards the catalyst's design

Achieving high selectivity and current density for C_2_ oxygenated products on pure Cu during CO_2_RR remains a significant challenge. The understanding of the oxygen-containing precursor coupling mechanism is thus substantial. We multiply the number of oxygen atoms involved in each coupling reaction by its coupling rate as the descriptor of the oxygen reaction rate (reaction rate of steps with oxygen-containing intermediate). The resulting oxygen reaction rate under different voltage and pH conditions was summarized and shown in Fig. 4c. Our results revealed a trend where the oxygen reaction rate increased with applied voltage, peaked at approximately −0.7 V *vs.* RHE, and then declined. By a similar approach, we extracted experimental current data from previous experiments by multiplying the current by the number of oxygen atoms in the products. As depicted in Fig. S33, our calculated reaction rates of oxygen-containing intermediates (this definition was introduced as a proxy for estimating the overall production tendency of oxygen-containing C_2_ products, under the simplifying assumption that downstream hydrogenation steps proceed at comparable rates. In other words, a higher oxygen-involving reaction rate indicates a higher formation rate of oxygen-rich intermediates, which are more likely to evolve into oxygen-containing final products such as ethanol or acetate.) closely matched the experimental results, showing the same trend of increasing and then decreasing with a peak at −0.7 V *vs.* RHE. Additionally, we calculated the ratio of oxygen reaction rates to hydrogen reaction rates under different pH and voltage conditions. Such a ratio only reached the same order of magnitude under pH 14 with no applied voltage and decreased rapidly with the pH decrease or voltage increase to zero. The comprehensive trend is illustrated in Fig. S34. *CO dominates the surface, and the hydrogenation process is greatly hindered without applied voltage, resulting in the highest oxygen content among intermediates. However, the reaction rate under these conditions is nearly zero, leading to an extremely low overall oxygen reaction rate. As the applied voltage increases, the hydrogenation of *CO intensifies, and the O/H ratio thus decreases. Concurrently, the reaction rate accelerates, driving the overall oxygen reaction rate upward until it peaks. Beyond this peak, although the reaction rate continues to increase, the oxygen content of the intermediates approaches zero. The rapid decrease rate of the oxygen content leads to a decline in the overall oxygen reaction. Such a peak in oxygen reaction rate occurs at −0.7 V *vs.* RHE.

Based on our study, such limitation stems from the intermediate and coupling mechanism (from carbon–oxygen to carbon–hydrogen–oxygen and eventually carbon–hydrogen intermediates) with the increase of the applied voltage. Under high applied voltage, the coupling between hydrocarbon intermediates becomes the primary reaction pathway, favoring the formation of C_2_ products such as ethylene over oxygenates like ethanol. This intrinsic property of Cu restricts its efficiency in producing high-value oxygenates. We also calculate the energy barrier of 20 coupling mechanisms, except *OC–CO coupling mechanisms, in Fig. S38, finding that the trend of C–C coupling barriers remains essentially the same under implicit and explicit solvation conditions, with only minor energy fluctuations observed. These variations are generally within approximately 0.2 eV, which falls within the typical energy uncertainty range of DFT calculations. This observation confirms the robustness of the newly proposed coupling mechanisms and highlights their relative insensitivity to solvation modeling artifacts, further supporting their potential applicability across diverse operating environments.

Number research thus explored strategies to modify the surface chemistry of Cu by introducing additional active sites by alloying with other metals like Ag or Pd.^26–28^ Ag and Pd are known for their substantial capacity to reduce CO_2_ to carbon monoxide with high selectivity. Thus, when alloying with Ag or Pd, such a synergistic effect can be achieved towards the selectivity to oxygenated products like ethanol by promoting the coupling of key intermediates. On the Ag sites, CO_2_ reduction stops at CO due to the weak binding of subsequent intermediates for further reduction. The *CH_2_ on the Cu surface will be generated at high potential. The proximity of CO(from weak adsorption metal) and hydrogen–carbon intermediates facilitates the coupling to form *H_2_C–CO intermediates, significantly protecting the oxygen from further attack by hydrogen, serving as a precursor to ethanol and other oxygenates. Such findings are remarkably consistent with the previous research, particularly Wang's work, where the FE of the ethanol increases with the ratio of Ag increases.^29^ This is because Cu facilitates the further reduction of oxygenation intermediates and plays no role in protecting the oxygen. These findings underline the importance of alloying strategies in tailoring the reaction environment on the catalyst surface. The introduction of Pd alters the local electronic structure and changes the intermediates' adsorption and reaction properties.^22,28,30,31^ Specifically, the alloying metals create a dual-site system that optimizes the adsorption energies of CO and hydrocarbon species, promoting the coupling reactions necessary for C_2_ oxygenate formation. Additionally, these modifications can suppress undesirable hydrogen evolution reactions (HER), which often compete with CO_2_RR on pure Cu. The success of Ag–Cu and Pd–Cu bimetallic catalysts highlights several design principles for developing high-performance systems for CO_2_RR.

The first is to ensure the uniform distribution of CO-generating sites like Ag and Pd near hydrocarbon-generating Cu sites. It allows for efficient intermediate coupling. The second is that the alloying element must be chosen to stabilize the intermediates, like *CO, which are precursors to C_2_ oxygenates. Higher voltages are typically required to achieve a high current. The hydrogenation process is an electrochemical step, and the reaction rate increases with increasing voltage. The oxygenated group is hard to preserve at high voltages. Therefore, to obtain the oxygen-containing products under high current conditions, the alloy element must incorporate metals like Ag and Pd, preventing the reaction from proceeding further in the hydrogenation in terms of the coupling mechanism.

## Conclusions

In this work, our study systematically discusses, for the first time, six distinct intermediates and all 21 possible coupling mechanisms under varying pH conditions and applied voltages. From a thermodynamic perspective, we analyzed how the hydrogenation process weakens the C–O bond and lowers the coupling barriers. Subsequently, we developed a microkinetic model to simulate the selectivity and activity of different products under pH 13, which aligns closely with previous experimental findings. By integrating surface coverage changes into our analysis, we concluded that the intermediates observed experimentally might not necessarily be the primary ones actively participating in the reaction. Additionally, we simulated the coupling mechanisms and surface coverage across various pH and voltage conditions, revealing the dynamic evolution of coupling pathways under different reaction environments. These insights provide valuable references for both experimental and computational researchers. Furthermore, we detailed and analyzed coupling mechanisms across different pH levels and voltages, illustrating the dynamic shifts in reaction pathways. Such shifts in the reaction pathways also provide valuable insights into the selectivity towards the oxygen-containing products. Our results demonstrate strong agreement with existing experimental findings and offer specific recommendations for enhancing the faradaic efficiency of different products in future studies. By linking coupling mechanisms to product formation, our work provides a comprehensive framework for understanding and optimizing CO_2_RR, paving the way for more efficient catalyst designs.

## Methods

### Density functional theory calculations

All density functional theory (DFT) calculations were performed using the Vienna *Ab initio* Simulation Package (VASP).^32,33^ The plane-wave basis set was employed with a kinetic energy cutoff of 450 eV, ensuring convergence of total energies. The exchange–correlation interactions were described using the Perdew–Burke–Ernzerhof (PBE) functional within the generalized gradient approximation (GGA).^34^ All symmetry operations were explicitly turned off to allow full structural relaxation without constraints. For the electronic smearing, Methfessel-Paxton first-order smearing was adopted with a width of 0.2 eV, suitable for metallic systems. The electronic self-consistency loop was considered converged when the total energy change between iterations fell below 10^−7^ eV, with at least 5 and at most 60 SCF iterations performed per ionic step. The electronic minimization was handled using the fast Davidson iteration scheme, and real-space projection operators were automatically selected to improve computational efficiency. Grimme's DFT-D3 dispersion corrections were included to account for van der Waals interactions.^35^ The Brillouin zone was sampled using a *Γ*-centered Monkhorst–Pack grid of 3 × 3 × 1, which was found sufficient to converge the total energy for the chosen surface model.^36^ We modeled the Cu(111) surface using a periodic slab consisting of four atomic layers in a (3 × 3) surface supercell. To mimic realistic surface conditions while minimizing computational cost, the bottom two atomic layers were fixed in their bulk positions, and only the top two layers were allowed to relax. This partial relaxation approach is commonly employed in surface calculations to reduce spurious slab polarization and simulate the semi-infinite bulk beneath the surface. The vacuum layer was set sufficiently large (typically >15 Å) to prevent spurious interactions between periodic images along the surface. Initial charge densities were generated by atomic superposition.

For the geometry optimization, implicit solvation effects were included using the VASPSOL model, with a surface tension-related cavity energy term set to 80 to express the aqueous environment.^37,38^ For the explicit solvation structure, water densities were chosen to be close to that of the Pt(111) bilayer structure as found in UHV experiments within the unit cell sizes considered.^39^ Water layer structures were determined using a minima-hopping algorithm that alternates between molecular dynamics and geometry optimization steps to construct a series of local minima.^40^ The geometry optimization settings used in the minima hopping procedure are consistent with those described above. To simulate a charged double layer at the electrochemical interface, a single hydrogen atom was placed in the water layer. The ground-state electronic structure redistributes the charge from this atom's one electron to the metal, creating a charge-separated double layer.^41^ All reaction-free energies were calculated based on DFT electronic energies corrected with zero-point energy (ZPE) and entropy contributions at 298.15 K. For each intermediate and transition state, vibrational frequency calculations were performed within the harmonic approximation using finite displacements of adsorbed atoms. The ZPE for each species was obtained by summing the zero-point contributions from all vibrational modes:1
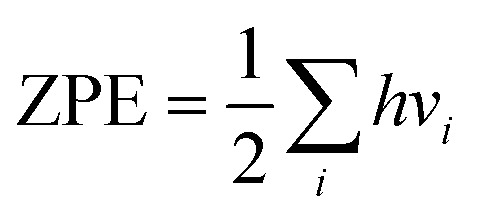
where *h* is the Planck's constant and *ν*_*i*_ is vibrational frequency *i* which is calculated based on the harmonic oscillator approximation. The standard molar vibrational thermal energy contribution is calculated by:2
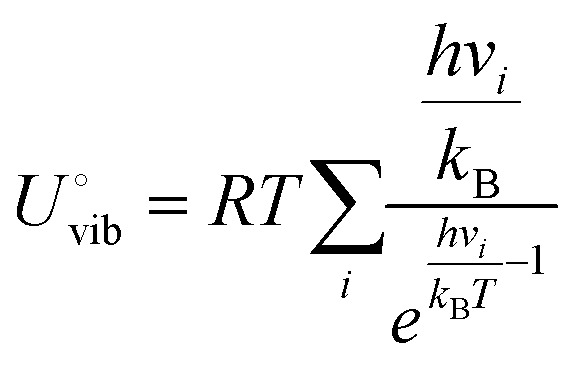
where *R* is the gas constant and *k*_B_ is Boltzmann's constant. The standard molar vibrational entropy is calculated using the following expression:3
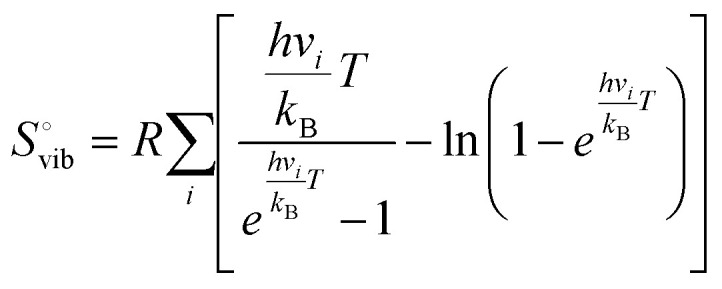


Imaginary frequencies (if any) associated with transition states were excluded from this summation.

The GC-DFT calculations were performed in JDFTx.^42^ We adopted the optimized structure from the VASP package and calculated the single-point energy under different potentials to test the reliability of our solvation model. The Brillouin zone was sampled using a uniform 3 × 3 × 1 Gamma-centered *k*-point mesh, suitable for surface slab geometries. Ultrasoft pseudopotentials from the GBRV library were employed for all elements.^43^ The Perdew–Burke–Ernzerhof (PBE) generalized gradient approximation was used for exchange–correlation interactions. A plane-wave kinetic energy cutoff of 20 Hartrees was applied. Electronic minimization was performed with a convergence threshold of 1 × 10^−5^ Hartree. Finite-temperature Fermi smearing of 0.1 eV was used. Implicit solvation was modeled using the CANDLE variant of the LinearPCM method with water as the solvent, and ionic concentrations of 0.1 M K^+^ and F^−^ were included.^44^ A target electron chemical potential was set, and van der Waals interactions were included. All calculations were spin-polarized along the *z*-axis.

### Gibbs energy and energy barrier calculations

We utilized the computational hydrogen electrode model to calculate reaction energies as a function of potential. At a potential of *U* = 0 V *versus* RHE, protons and electrons are in equilibrium with hydrogen gas (H_2_) under standard conditions applicable across all pH levels:^45^

4 H^+^ + e^−^ → H_2(g)_

At a given U≠ 0 V *versus* RHE,5
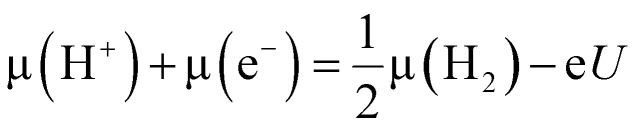


For calculating the Gibbs energy change of the energy barrier. To evaluate the effect of applied potential on proton-coupled electron transfer (PCET) steps, we employed a symmetry factor (*β*) of 0.5.^46^ This value reflects the assumption that the transition state involves the transfer of half an electron, which is commonly adopted in electrochemical kinetics when the charge transfer is concerted but the exact charge distribution at the transition state is unknown. Accordingly, the potential-dependent correction to the reaction barrier (Δ*G*^‡^(*U*)) was introduced as:

6 ΔG^‡^(*U*) = ΔG^‡^(0) − *β*e*U*

where Δ*G*^‡^(0) is the activation barrier at the reference potential (typically 0 V *vs.* RHE), *e* is the elementary charge, and *U* is the applied electrode potential. This correction effectively accounts for the electrostatic stabilization of the transition state in response to increasing overpotential. The use of *β* = 0.5 ensures a balanced treatment of the potential influence, consistent with prior theoretical treatments of PCET processes in electrocatalysis.^14,47–49^ In addition, in microkinetic modeling, we directly represent the influence of pH on the overall reaction kinetics through the concentration of proton donors or acceptors (*e.g.*, H^+^), which are modulated in accordance with the pH value.

### Microkinetic modeling

In microkinetic modeling, the reaction network is constructed from a series of elementary steps, and fundamental thermodynamic and kinetic principles govern the net rate of each step. According to the De Donder relation, the net rate *r*_*i*_ for an elementary step *i* can be expressed as a product of the forward rate constant *k*_*i*_. The surface coverages *θ*_*j*_ of the participating reactants, their stoichiometric coefficients *v*_*ij*_, and a reversibility factor (1 − *Z*_*i*_), where *Z*_*i*_ represents the thermodynamic driving force for reversibility.^50^ Mathematically, this is written as:7
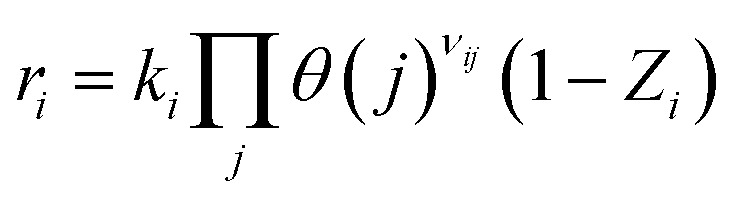
Here, 
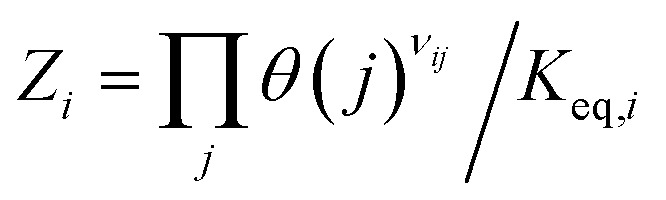
, where *K*_eq,*i*_ is the equilibrium constant of step *i*, given by8
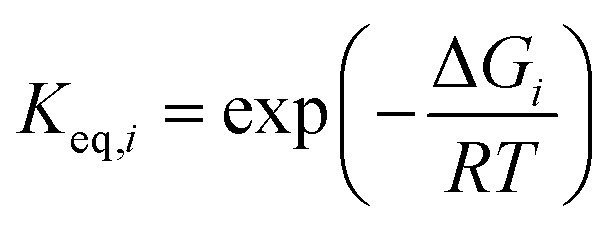


This term reflects the extent to which the step is reversible: *Z*_*i*_ → 0 for irreversible steps and *Z*_*i*_ → 1 for steps approaching equilibrium. The stoichiometric coefficient *v*_*ij*_ indicates the number of molecules of species *j* involved in step *i*.

In terms of input conditions, the model operates under room temperature. The partial pressures of CO were set as 10^−7^ bar products are set to be 10^−20^ bar, to describe the reaction condition at the beginning of the reaction. Mass transport limitations were not included in the simulations. The proton concentration was limited by setting the maximal proton concentration near the surface to 10^−pH^.

The Tafel slopes for C_2_H_4_, CH_4_, and H_2_ formation were determined based on turnover frequency (TOF) calculations at different applied potentials (*versus* the standard hydrogen electrode, SHE). Each TOF value was referenced to a model surface, where a 3 × 3 Cu (111) unit cell was considered to contain one active site. The number of active sites per unit area (1 cm^2^) was estimated by calculating the surface atom density of Cu (111). The total molecular formation rate per unit area was obtained by multiplying the TOF by the number of active sites per cm^2^. To convert the molecular formation rate into current density, the number of electrons transferred per molecule (12 for C_2_H_4_, 8 for CH_4_, and 2 for H_2_) and the elementary charge of an electron (1.602 × 10^−19^) were used. The resulting partial current density for each product was plotted against the applied potential on a logarithmic scale (log *j vs.* potential), and the Tafel slope was extracted by linear fitting in the kinetically controlled region.

## Author contributions

Z. W. supervised the project. C. Z. performed the work.

## Conflicts of interest

The authors declare no competing financial interest.

## Supplementary Material

SC-OLF-D5SC05367F-s001

## Data Availability

The data supporting this article have been included as part of the supplementary information (SI). Supplementary information: SI present detailed coupling rate and surface coverage distributions under different voltages and pH values, comparative analysis between experimental measurements and modeling results, JDFTx-based computational validation tests, and comprehensive information on all associated reaction pathways. See DOI: https://doi.org/10.1039/d5sc05367f.
