# Supplementary material for: Voltage- and pH-driven evolution of multi-pathway C–C coupling in CO2 electroreduction on copper
Source: Chem Sci. 2025 Oct 6;16(44):20978–89. doi: 10.1039/d5sc05367f (PMC12516213; doi:10.1039/d5sc05367f)
Supplement: SC-016-D5SC05367F-s001 [file SC-016-D5SC05367F-s001.pdf]

Supplementary Information (SI) for Chemical Science.
This journal is © The Royal Society of Chemistry 2025

Supplementary Information

Voltage- and pH-Driven Evolution of Multi-Pathway C-C Coupling in $CO_{(2)}$ Electroreduction on Copper

Chengyi Zhang,[a] Ziyun Wang*[a]

[a]School of Chemical Sciences, University of Auckland, Auckland, New Zealand

Correspondence: ziyun.wang@auckland.ac.nz

Table of Contents

1. Insensitivity to Computational Advancements 3

2. Supporting Tables and Figures..5

3. Supporting References 28

1. Insensitivity to Computational Advancements

We carried out GC-DFT calculations using the JDFTx package, examining how the coupling barrier changes with increasing applied potential versus the standard hydrogen electrode (SHE). The applied potentials ranged from 0.0 to –0.9 V versus the reversible hydrogen electrode (RHE), corresponding to 0.0 to –1.8 V versus SHE. For this analysis, single-point energy calculations under different potentials were performed using JDFTx on geometries previously optimized with the VASP package.[1] As shown in Fig. S35, the deviation of the *OC-CO coupling barrier relative to our previously reported values is generally within 0.2 eV, which falls within the typical error margin of standard DFT calculations. Moreover, our study highlights the competition among multiple C-C coupling pathways beyond the conventional *OC-CO mechanism, thereby offering a broader framework that is not confined to a single reaction path or computational approach. To explore this, we conducted a sensitivity analysis in which all other coupling barriers were held constant, while the *OC-CO coupling barrier was artificially reduced to an unrealistically low value of 0.4 eV. Although this scenario is unlikely given that the Volmer step barrier has been reported as ($0.42 \times U_{SHE} + 1.43$),[2] or 0.8 eV in previous research[3] while observed pH-independence of C_{2+} product formation rates on the SHE scales indicates that proton transfer is not involved in the rate-limiting step. Under these hypothetical conditions, assuming *OC-CO coupling becomes rate-determining, the C_{2+} production rate would exceed that of the hydrogen evolution reaction (HER) at modest overpotentials. However, experimental observations consistently show HER dominance at low overpotentials in CO reduction

experiments. To further investigate this, we employed microkinetic modeling at pH 13 with the *OC-CO coupling barrier set to 0.4 eV. The results are shown in Fig. S36, which shows that the multi-coupling mechanisms are not changed. Interestingly, even under this highly biased assumption, our results still show the emergence and dynamic evolution of multiple coupling pathways with increasing potential. These findings strongly support the robustness and reliability of our proposed multi-path coupling mechanism. Due to the complexity and variability of realistic electrochemical environments, it is not feasible to accurately capture the exact energy barriers of every elementary step. Moreover, these barriers are likely to fluctuate dynamically under operating conditions. Nevertheless, our simulations robustly demonstrate the existence and dynamic evolution of multiple coupling pathways and their potential-dependent competition. It is also important to acknowledge the limitations of our microkinetic modeling. First, in all our density functional theory (DFT) calculations, we adopted an adsorbate coverage of 1/9 monolayer (ML) to evaluate the adsorption energies and activation barriers for the various intermediates and transition states. This corresponds to a relatively low surface coverage, which minimizes lateral interactions and is commonly used in electrocatalysis simulations. Importantly, recent experimental work using surface-enhanced infrared spectroscopy under high-pressure conditions (up to 60 barg) has shown that the saturation CO coverage on Cu surfaces under CO reduction reaction (CORR) conditions is approximately 0.25 ML.[4] This finding strongly suggests that CO poisoning is not a limiting factor under typical operating conditions and supports the physical plausibility of using low-coverage models in mechanistic studies.

While our microkinetic simulations were carried out using the CATKINAS software package, it should be noted that CATKINAS does not currently incorporate explicit adsorbate–adsorbate interactions. As a result, the coverages reported in our simulations may be somewhat overestimated. However, given the relatively low intrinsic coverage observed experimentally and our use of dilute-limit adsorption energies, we believe our model provides a reasonable and meaningful description of the key reaction trends and kinetics in CORR. Second, proton–electron transfers are treated within the computational hydrogen electrode (CHE) framework, which assumes a fixed proton donor and does not capture changes in proton donor identity across different pH conditions. Additionally, the mean-field approximation used in the model ignores spatial heterogeneity and electric double-layer effects, which can influence local concentrations and reaction kinetics. While these simplifications allow for efficient simulation and qualitative mechanistic insights, they may limit the quantitative accuracy of pH and potential-dependent trends.

2. Supplementary Tables and Figures

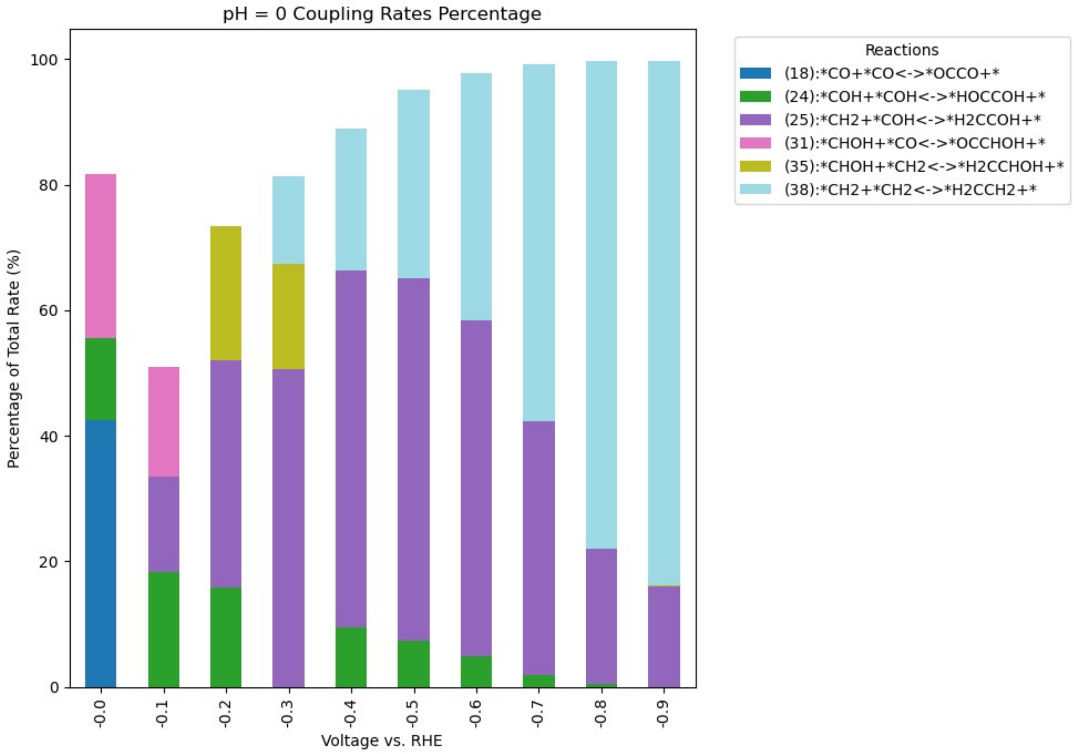

Fig. S1 The ratio of the top 3 coupling reactions to the total coupling reaction at pH 0.

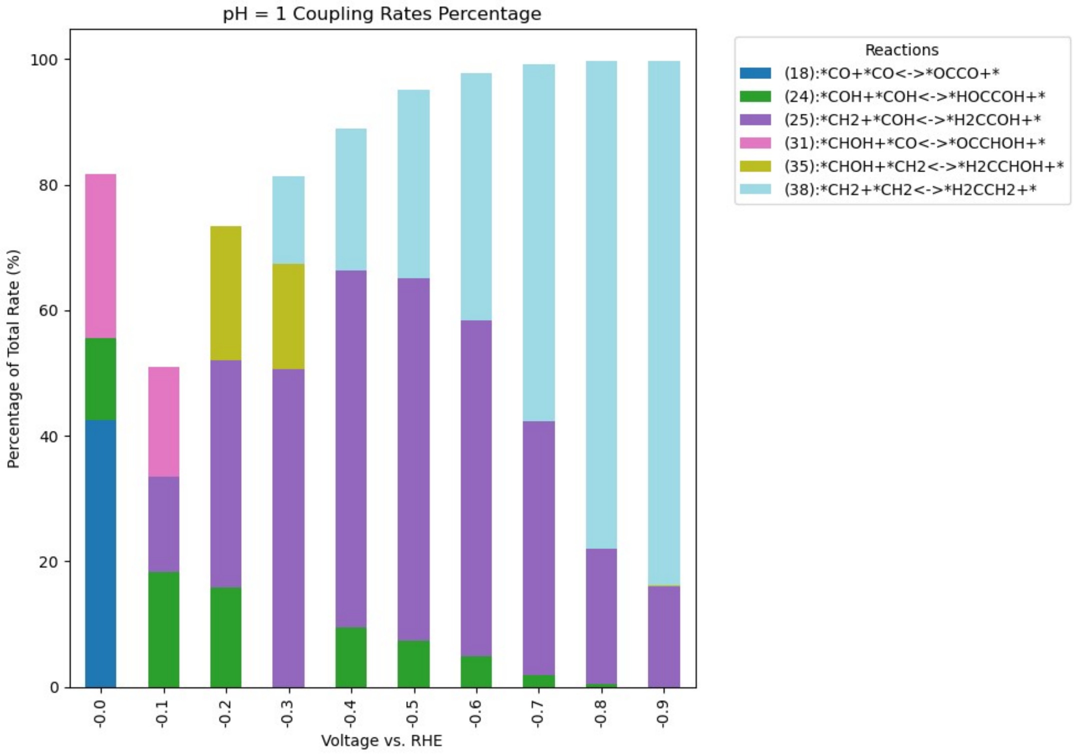

Fig. S2 The ratio of the top 3 coupling reactions to the total coupling reaction at pH 1.

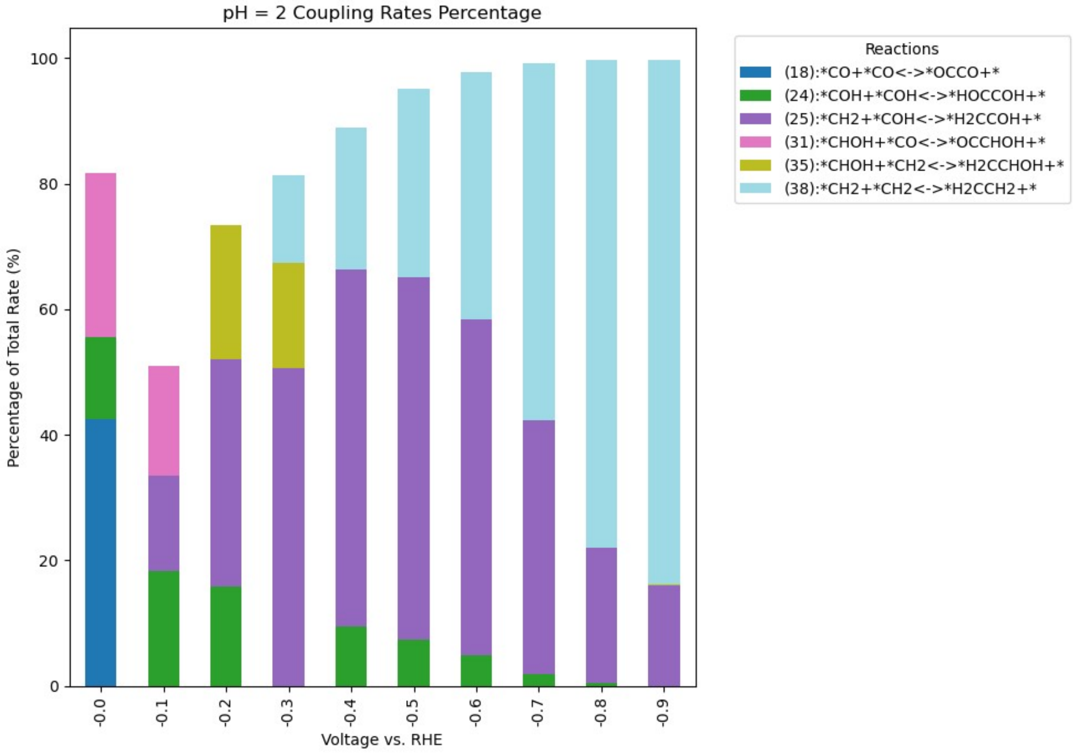

Fig. S3 The ratio of the top 3 coupling reactions to the total coupling reaction at pH 2.

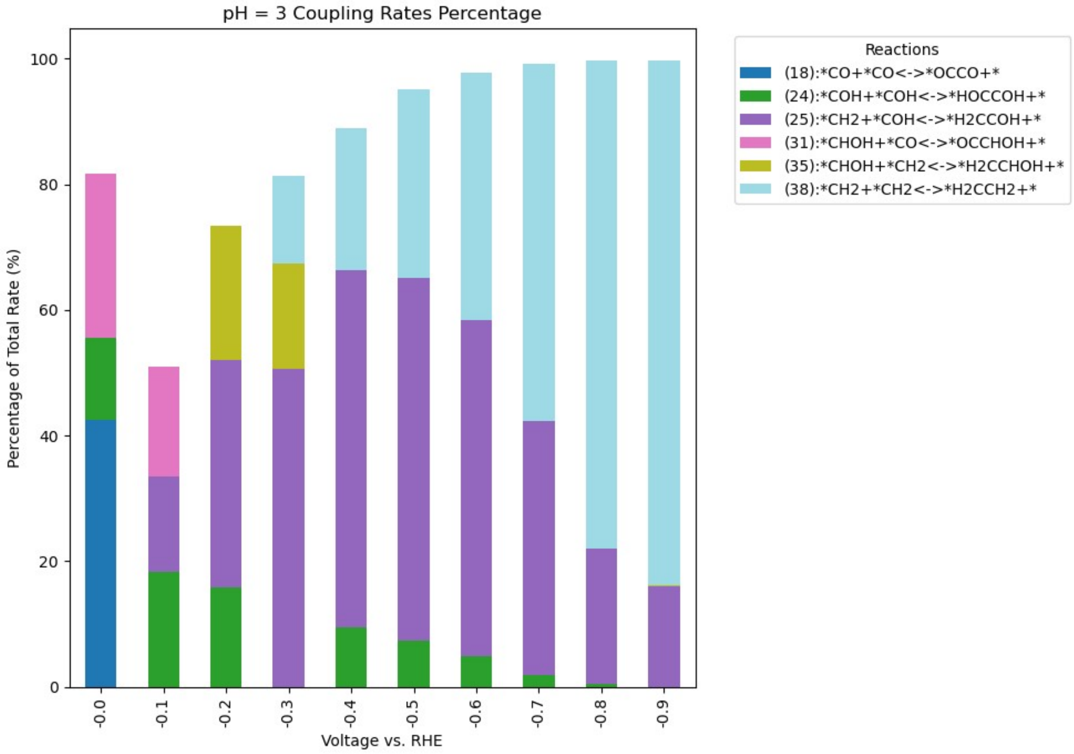

Fig. S4 The ratio of the top 3 coupling reactions to the total coupling reaction at pH 3.

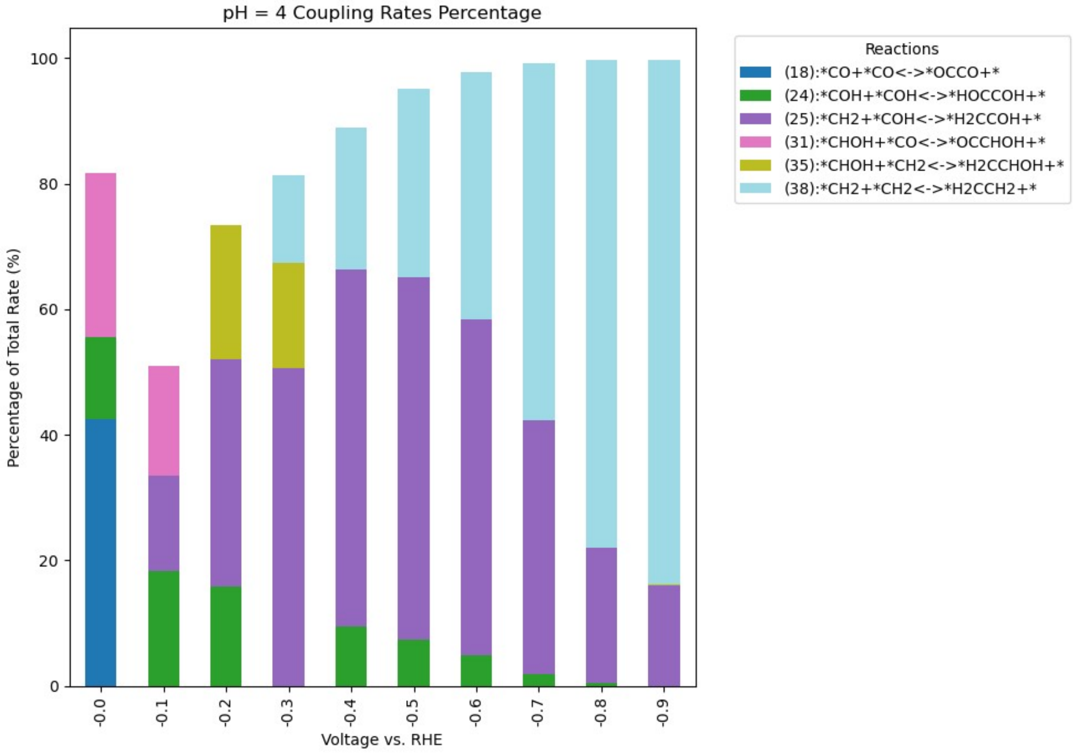

Fig. S5 The ratio of the top 3 coupling reactions to the total coupling reaction at pH 4.

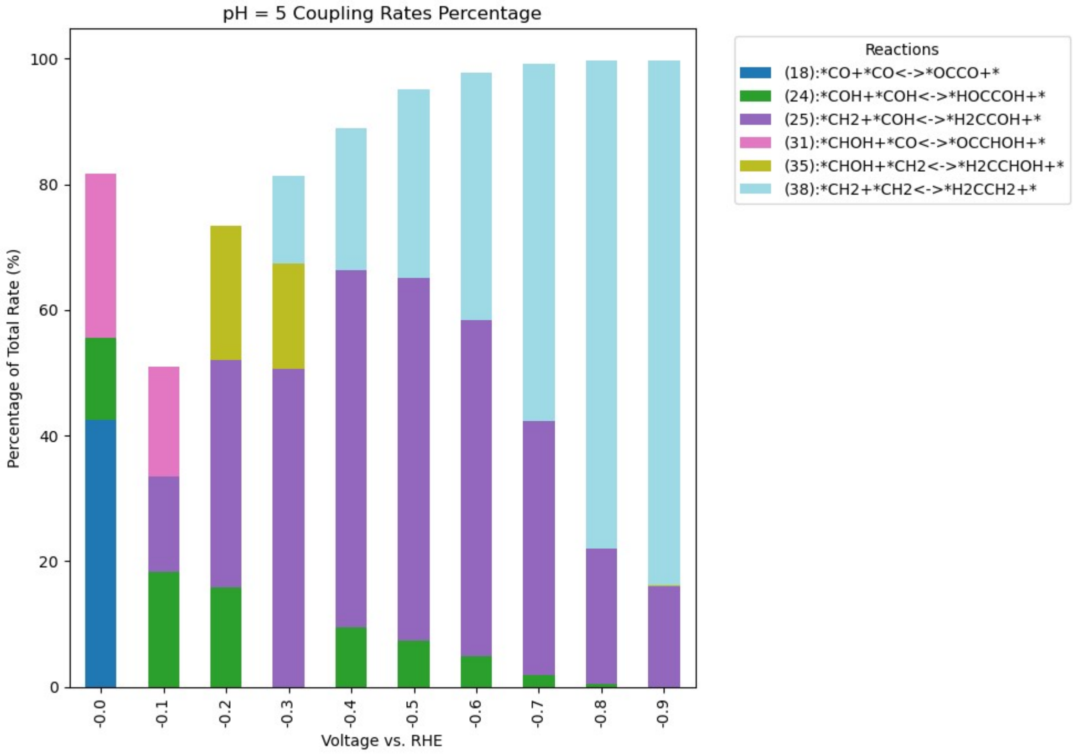

Fig. S6 The ratio of the top 3 coupling reactions to the total coupling reaction at pH 5.

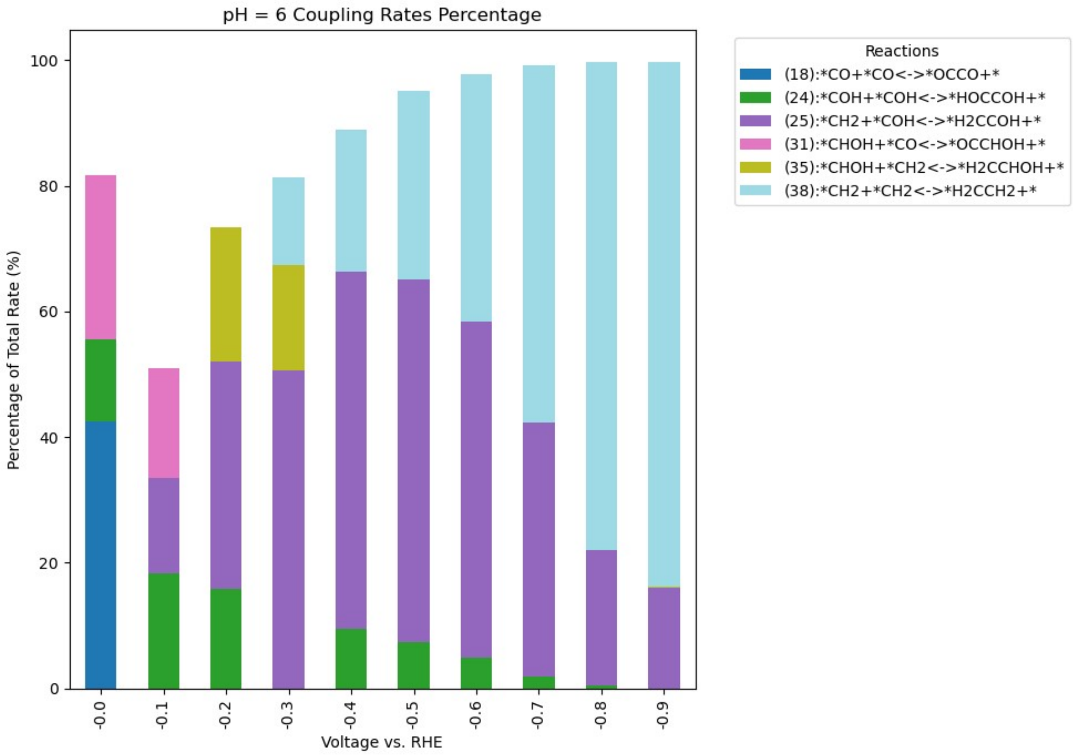

Fig. S7 The ratio of the top 3 coupling reactions to the total coupling reaction at pH 6.

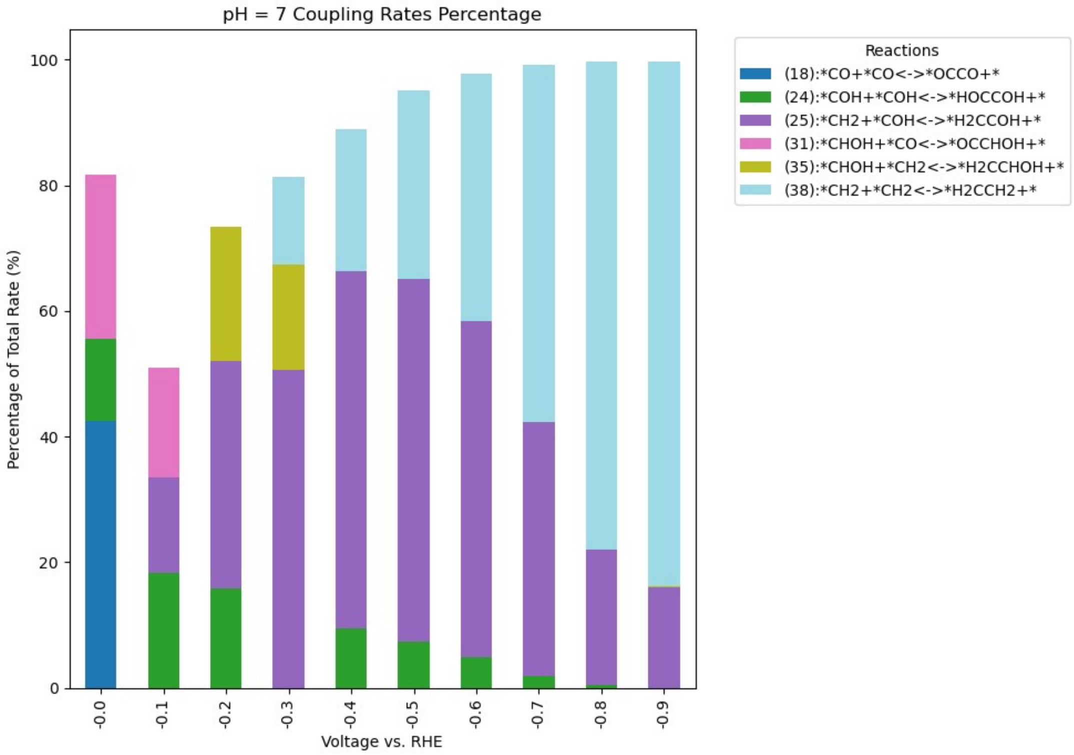

Fig. S8 The ratio of the top 3 coupling reactions to the total coupling reaction at pH 7.

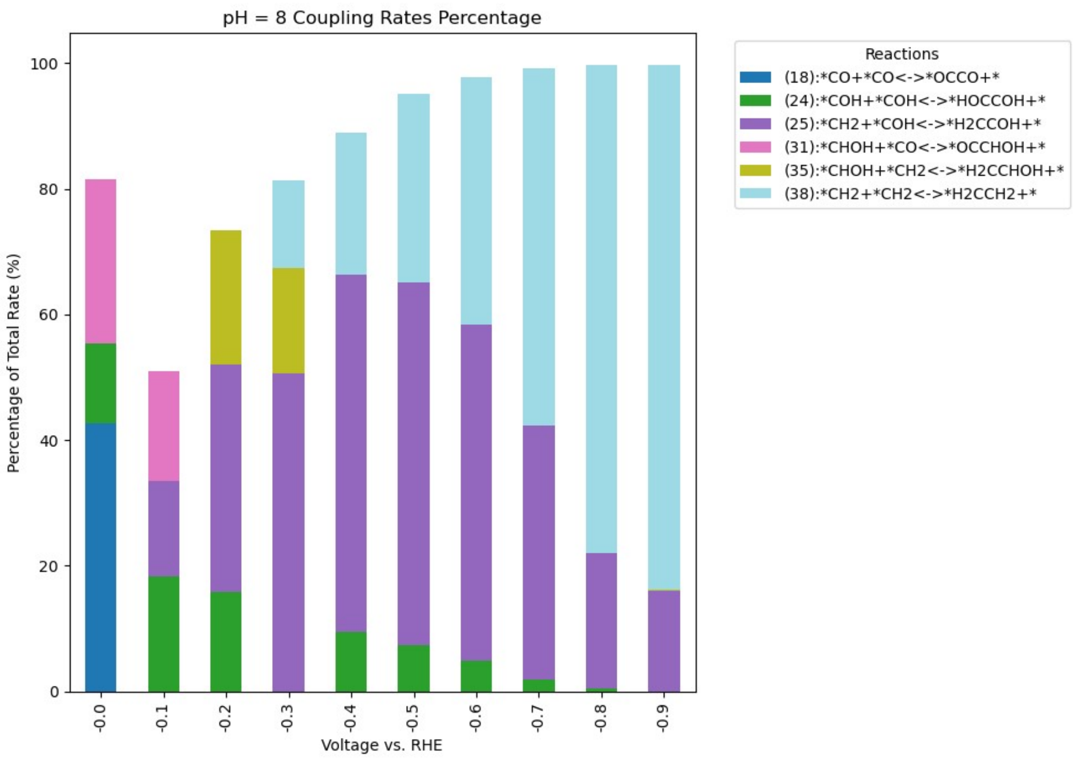

Fig. S9 The ratio of the top 3 coupling reactions to the total coupling reaction at pH 8.

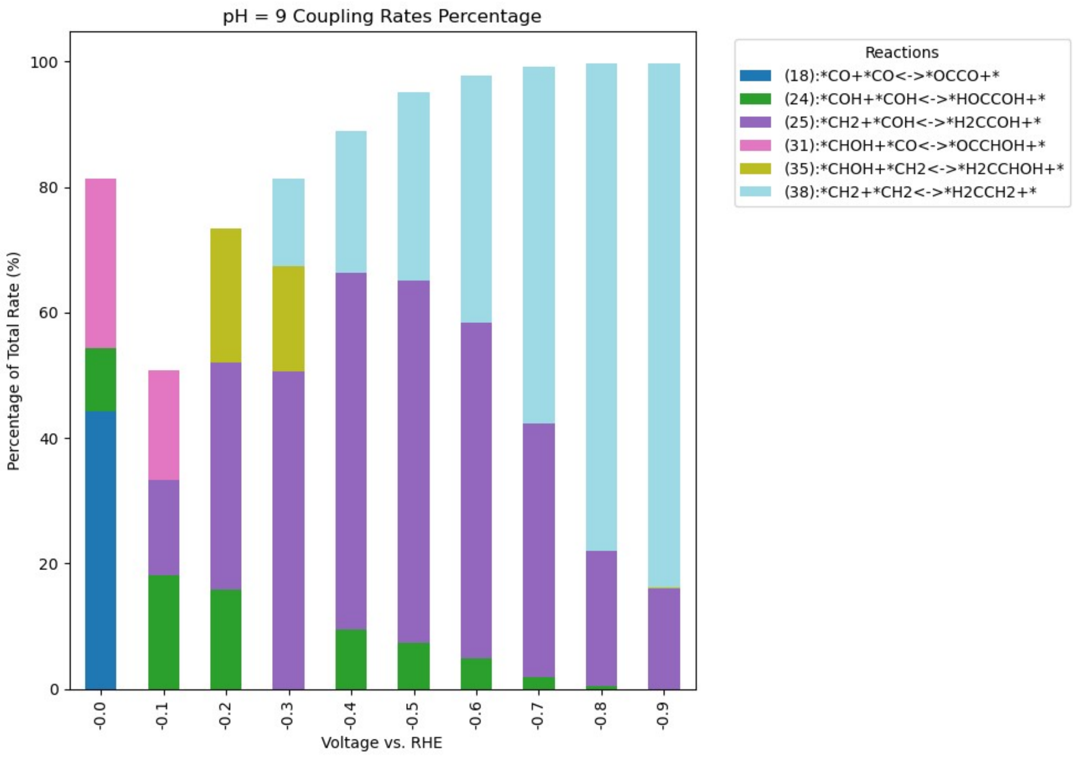

Fig. S10 The ratio of the top 3 coupling reactions to the total coupling reaction at pH 9.

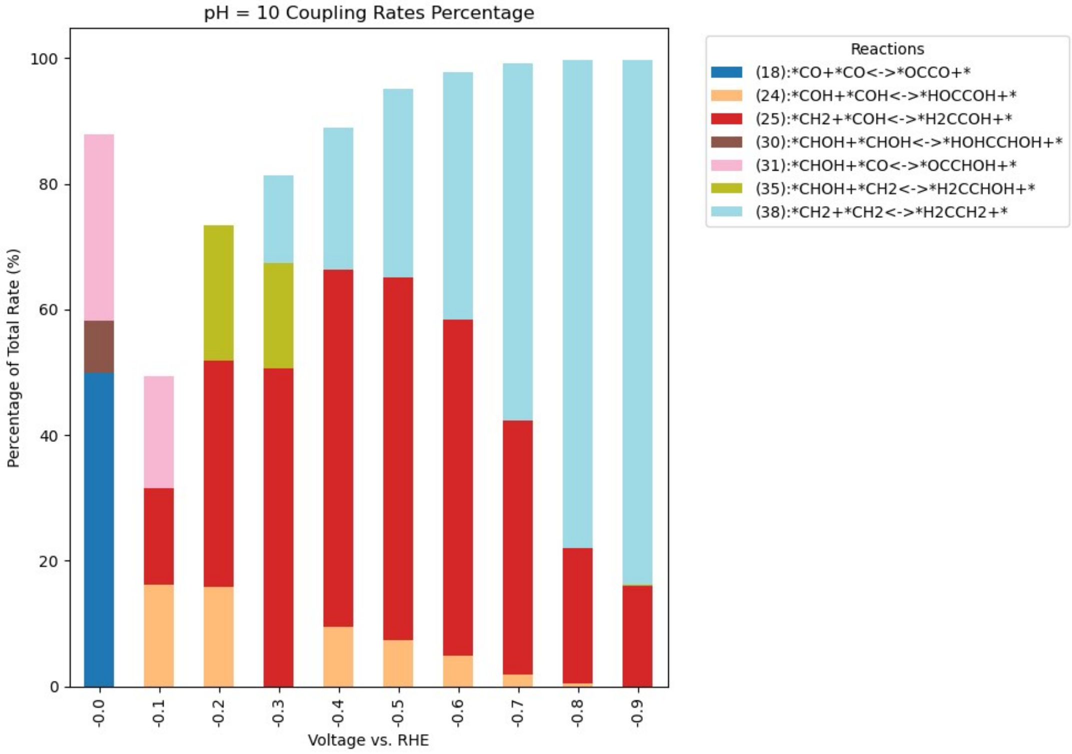

Fig. S11 The ratio of the top 3 coupling reactions to the total coupling reaction at pH 10.

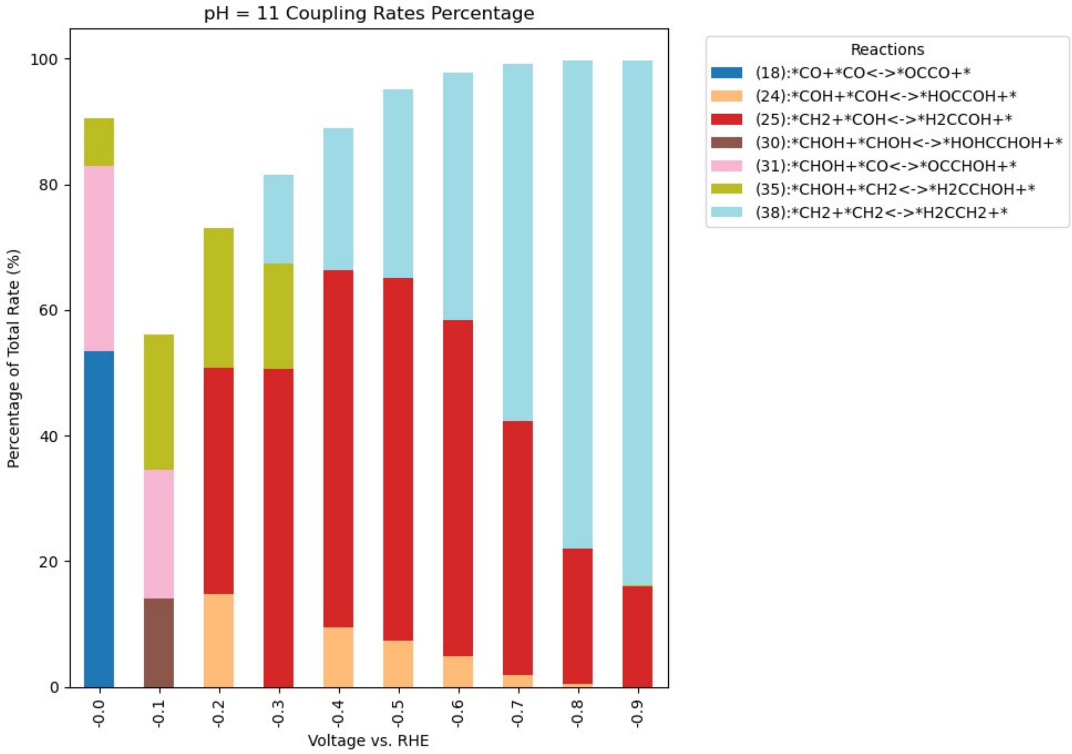

Fig. S12 The ratio of the top 3 coupling reactions to the total coupling reaction at pH

11.

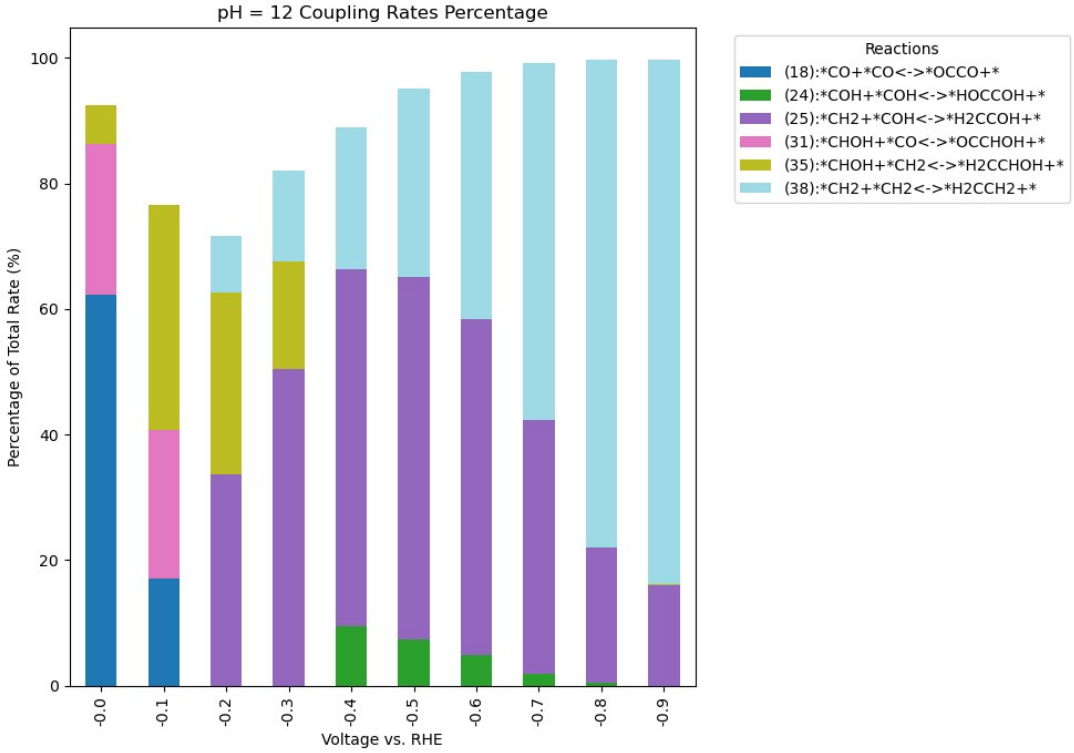

Fig. S13 The ratio of the top 3 coupling reactions to the total coupling reaction at pH 12.

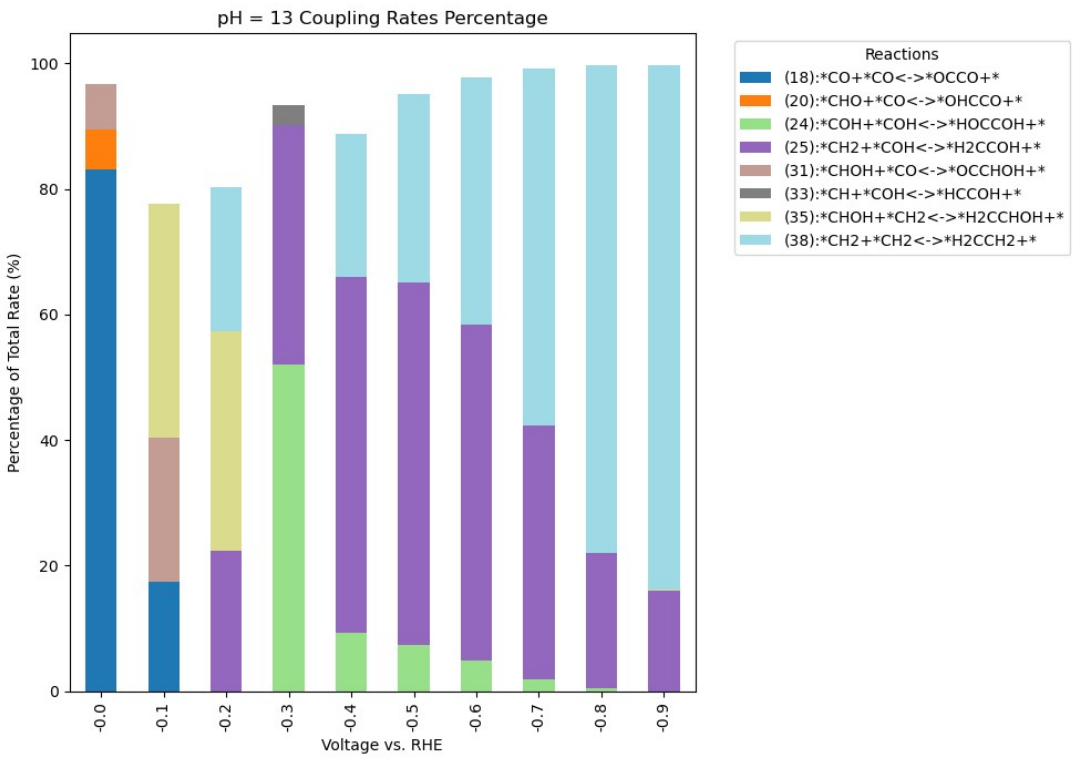

Fig. S14 The ratio of the top 3 coupling reactions to the total coupling reaction at pH 13.

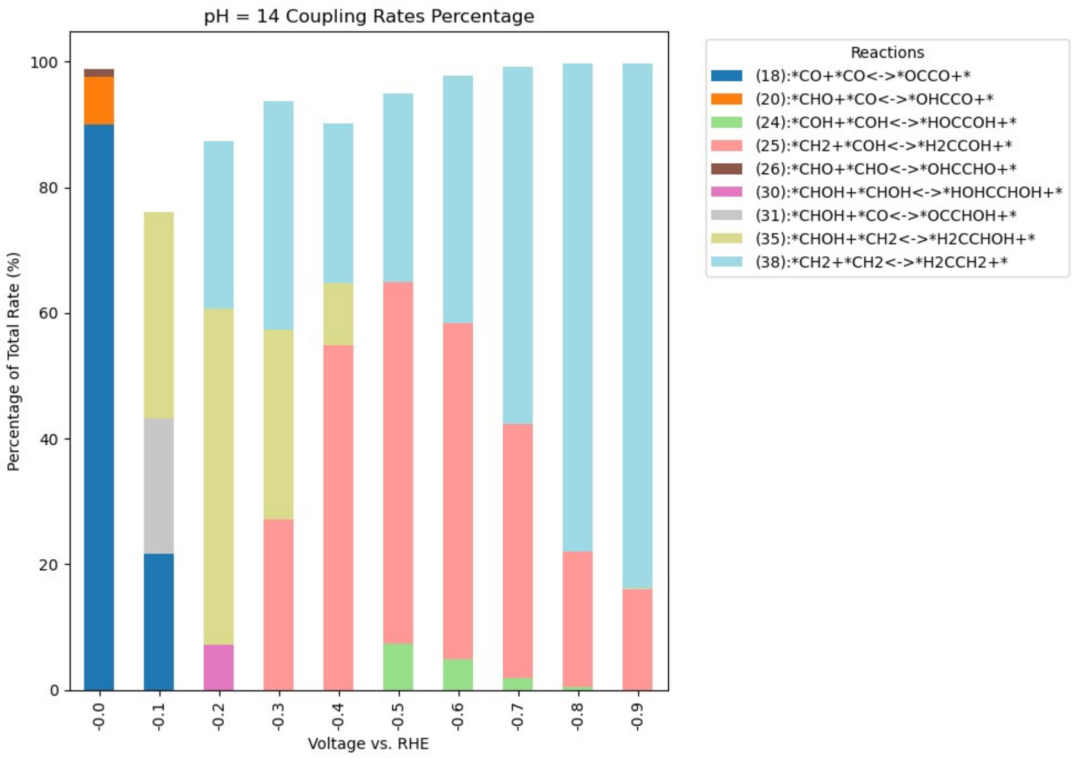

Fig. S15 The ratio of the top 3 coupling reactions to the total coupling reaction at pH

14.

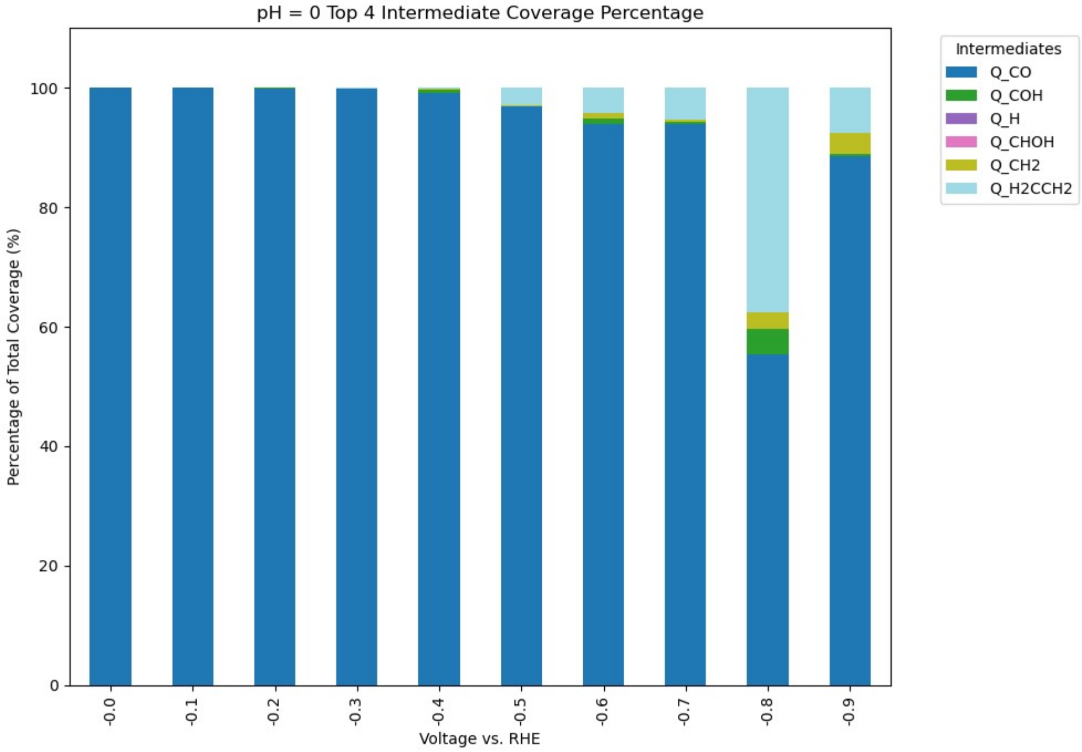

Fig. S16 The coverage ratio of different intermediates of different voltage on Cu(111) surface at pH 0.

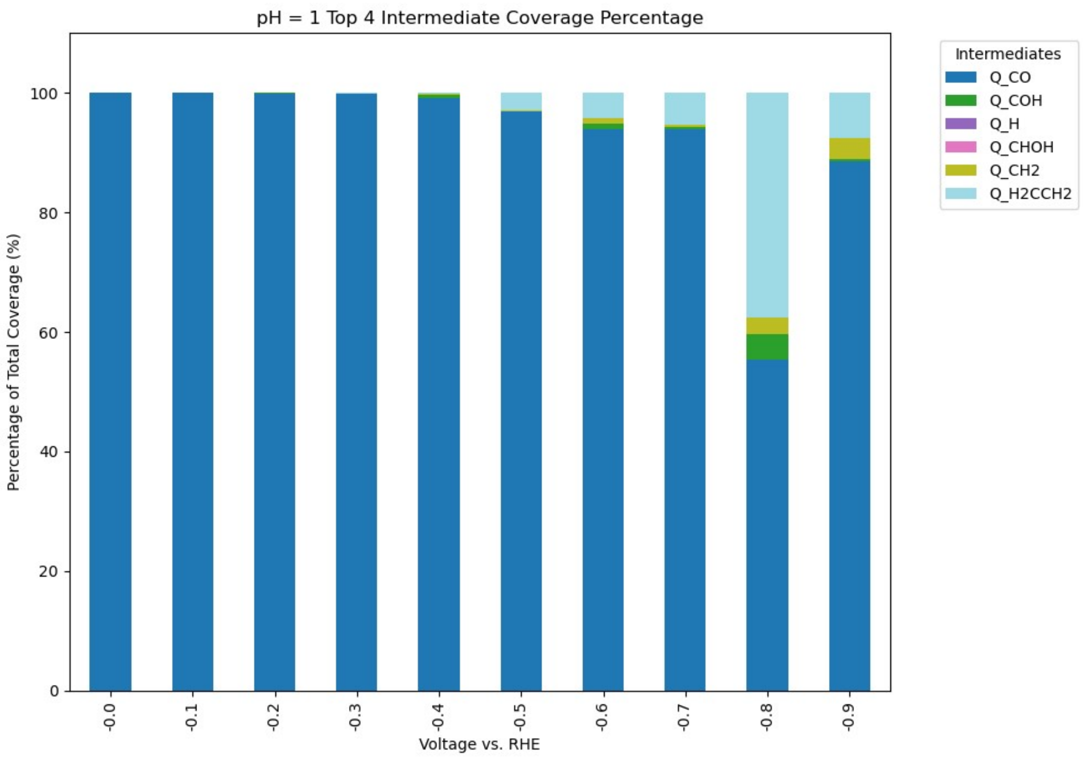

Fig. S17 The coverage ratio of different intermediates of different voltage on Cu(111) surface at pH 1.

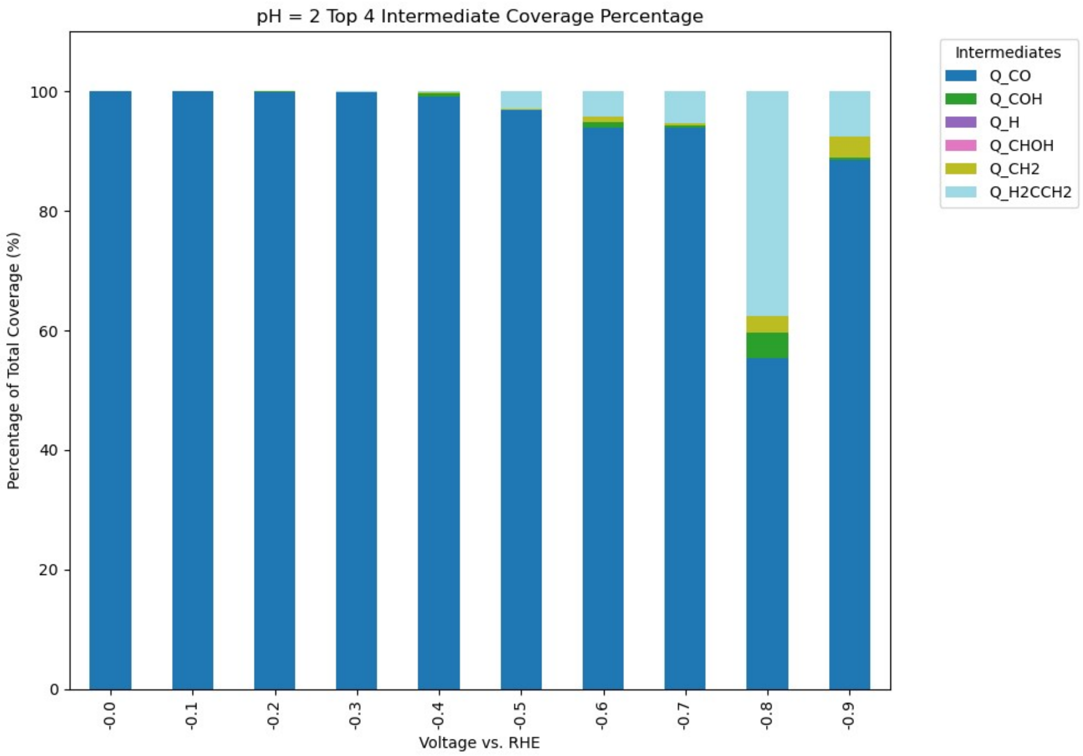

Fig. S18 The coverage ratio of different intermediates of different voltage on Cu(111)

surface at pH 2.

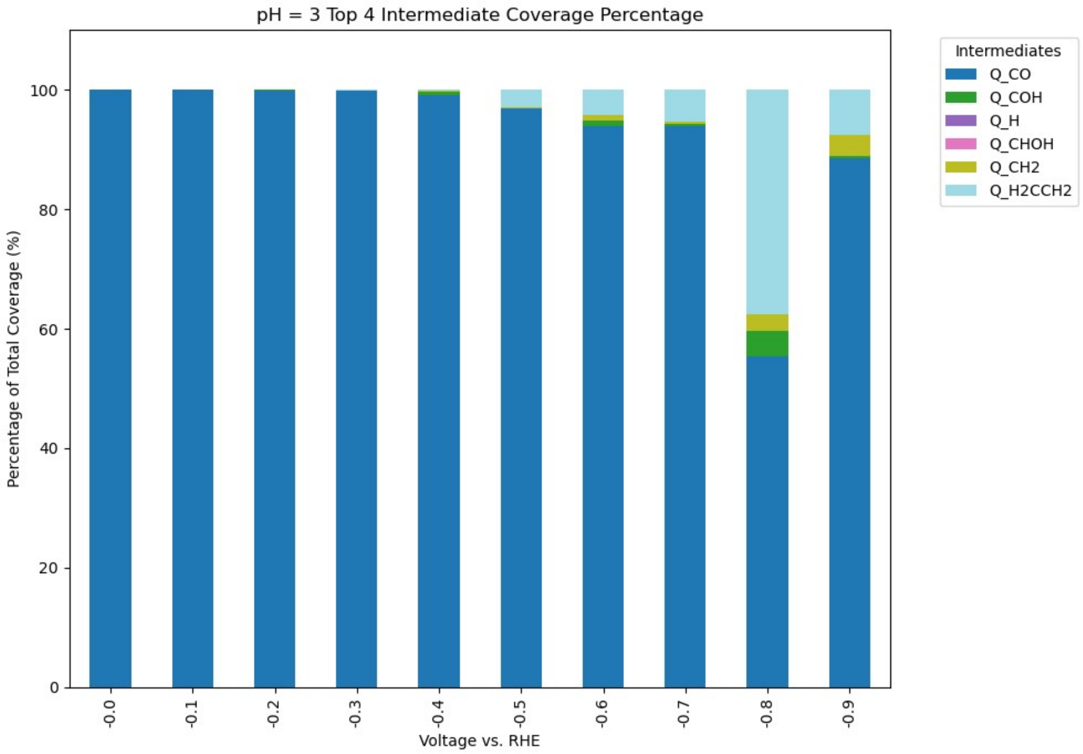

Fig. S19 The coverage ratio of different intermediates of different voltage on Cu(111) surface at pH 3.

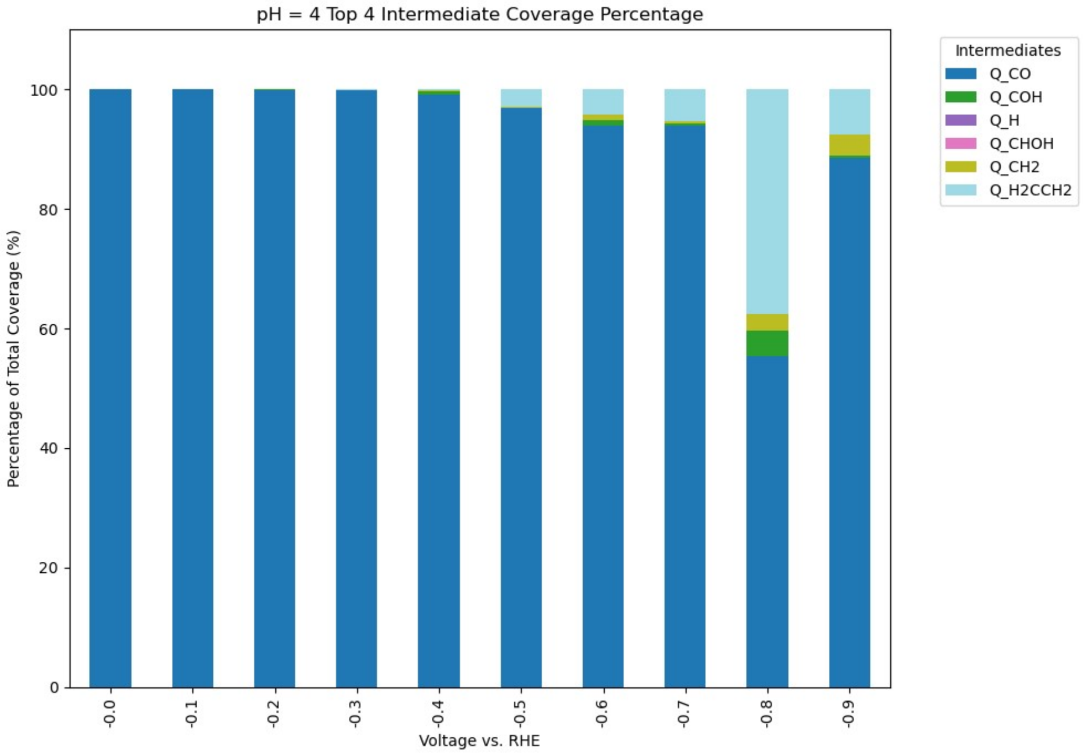

Fig. S20 The coverage ratio of different intermediates of different voltage on Cu(111) surface at pH 4.

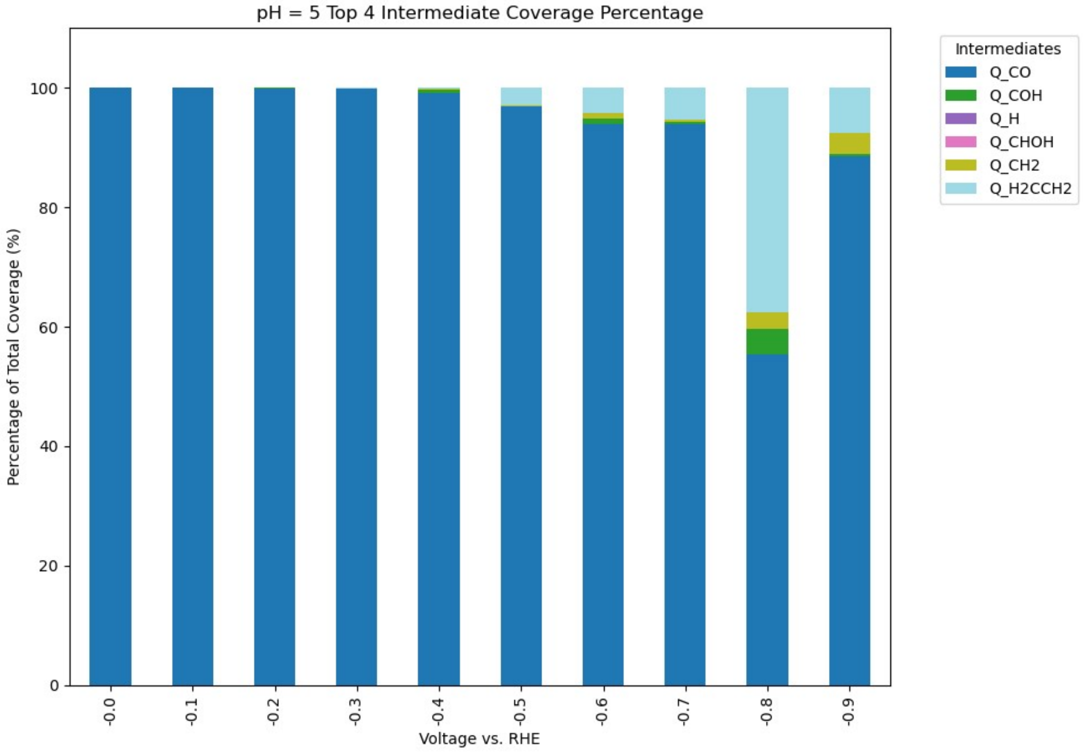

Fig. S21 The coverage ratio of different intermediates of different voltage on Cu(111) surface at pH 5.

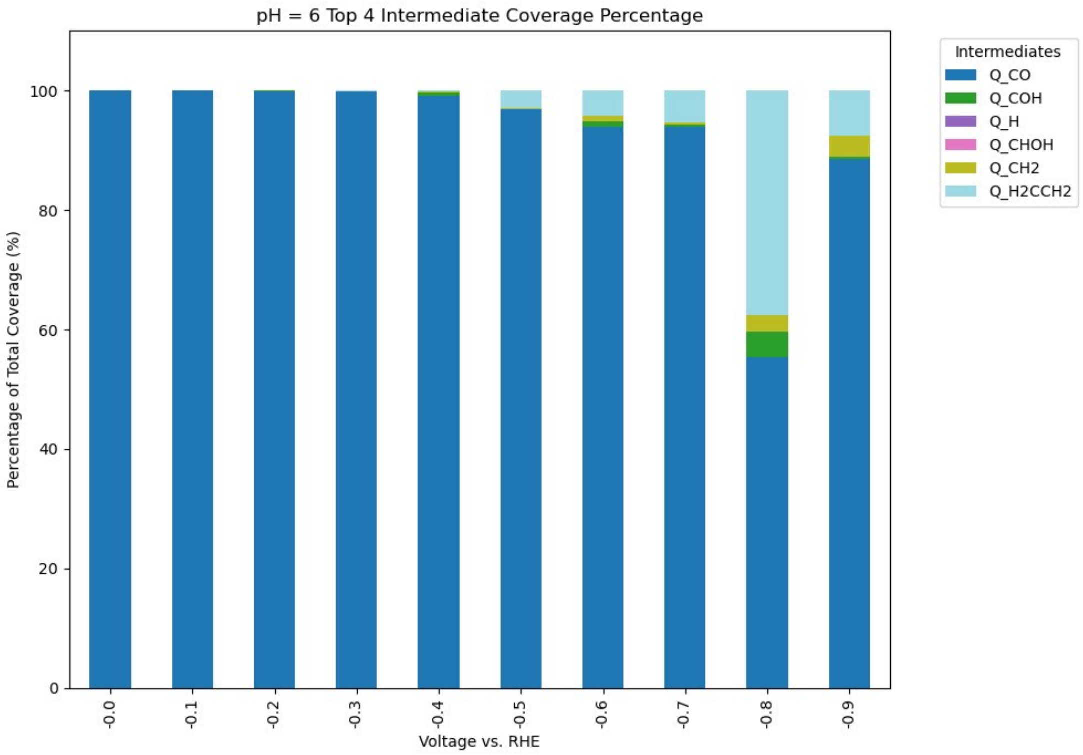

Fig. S22 The coverage ratio of different intermediates of different voltage on Cu(111) surface at pH 6.

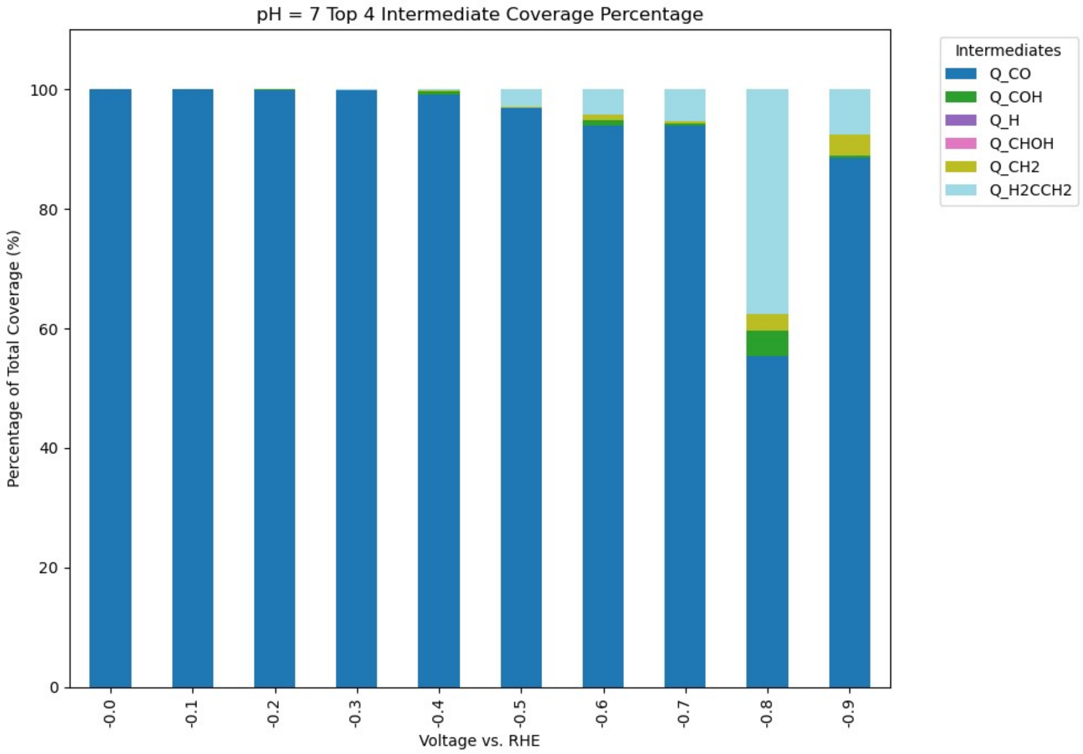

Fig. S23 The coverage ratio of different intermediates of different voltage on Cu(111)

surface at pH 7.

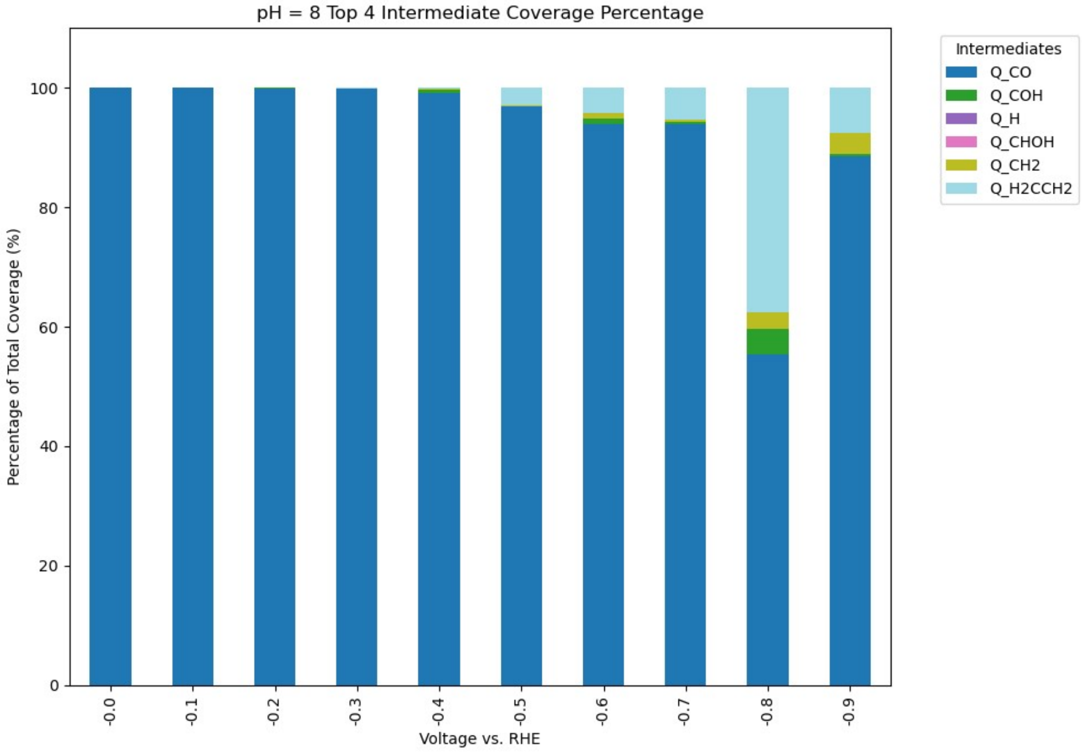

Fig. S24 The coverage ratio of different intermediates of different voltage on Cu(111) surface at pH 8.

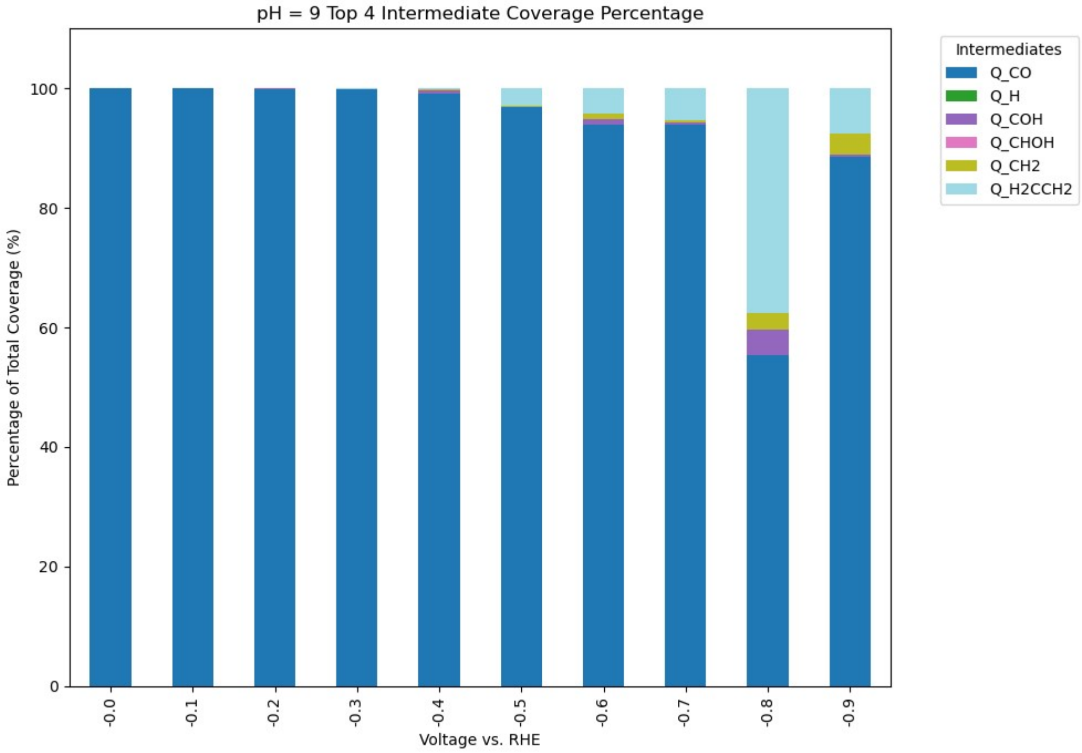

Fig. S25 The coverage ratio of different intermediates of different voltage on Cu(111) surface at pH 9.

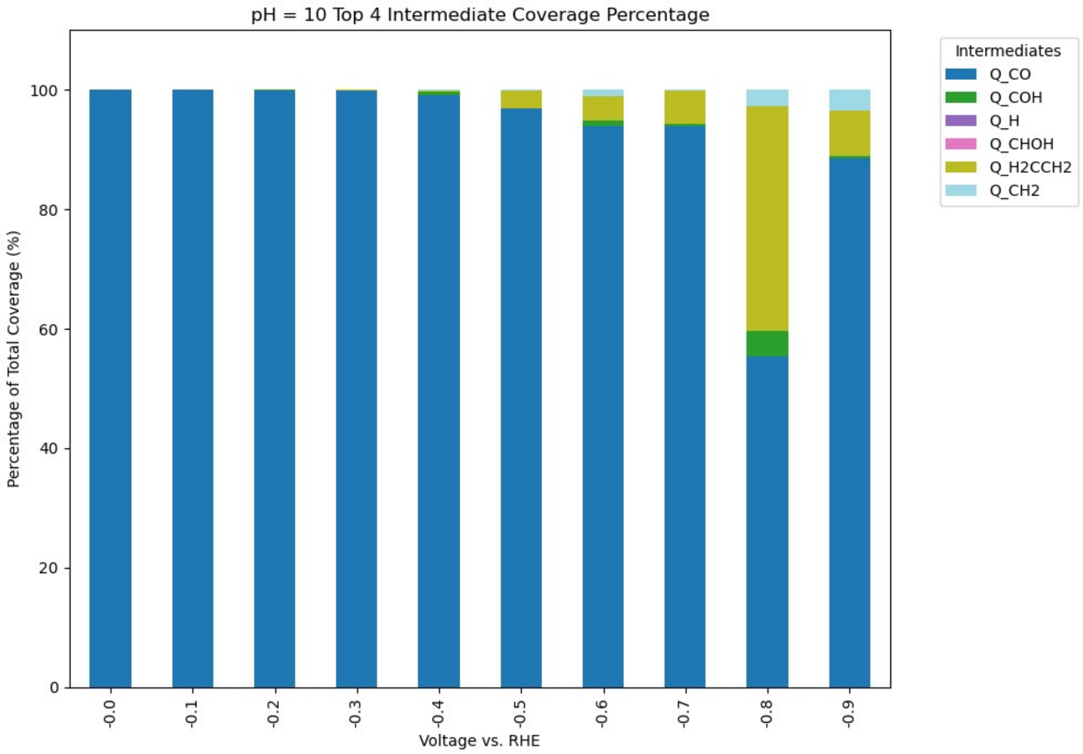

Fig. S26 The coverage ratio of different intermediates of different voltage on Cu(111) surface at pH 10.

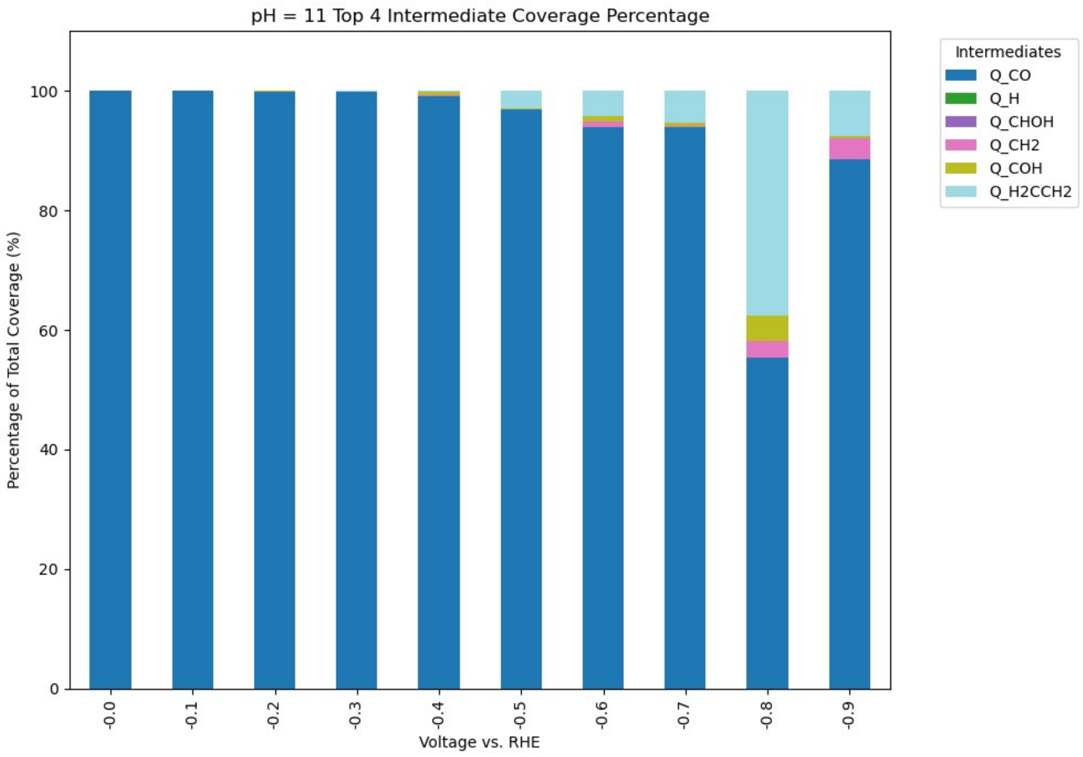

Fig. S27 The coverage ratio of different intermediates of different voltage on Cu(111) surface at pH 11.

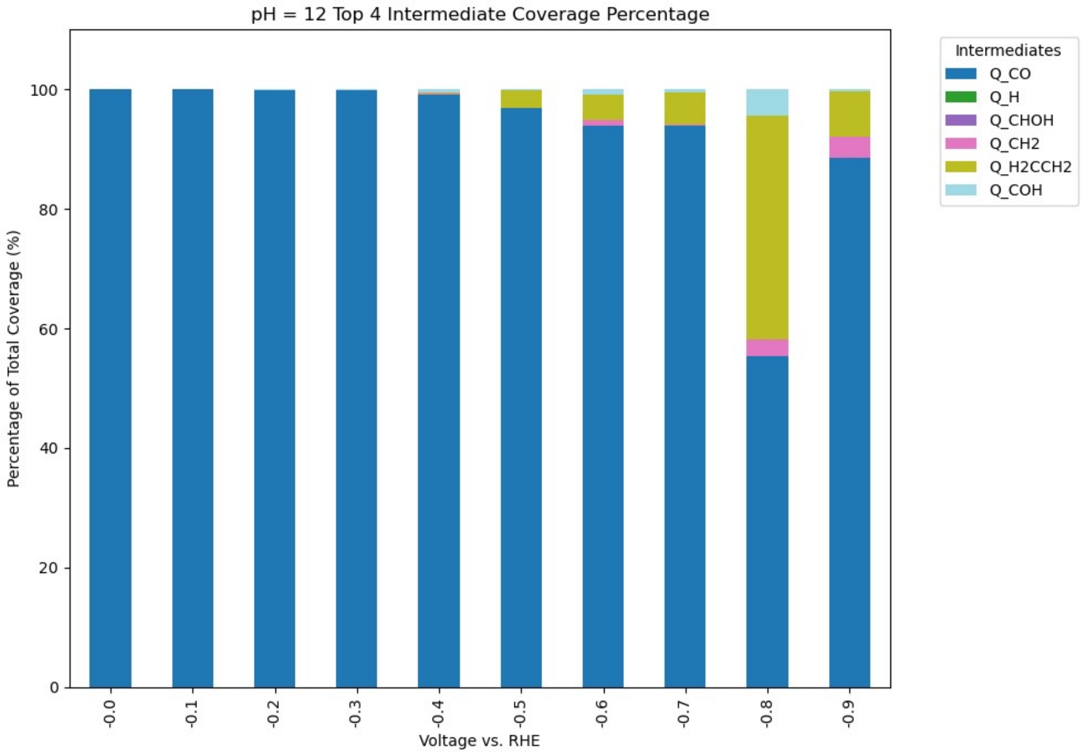

Fig. S28 The coverage ratio of different intermediates of different voltage on Cu(111)

surface at pH 12.

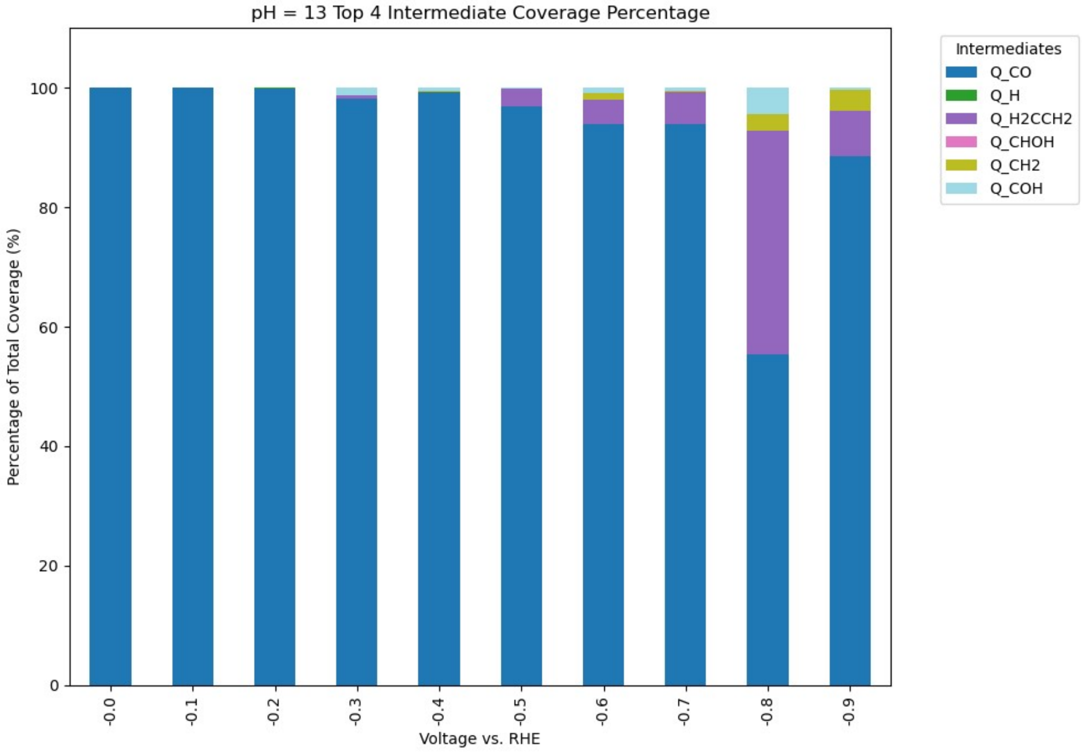

Fig. S29 The coverage ratio of different intermediates of different voltages on Cu(111) surface at pH 13.

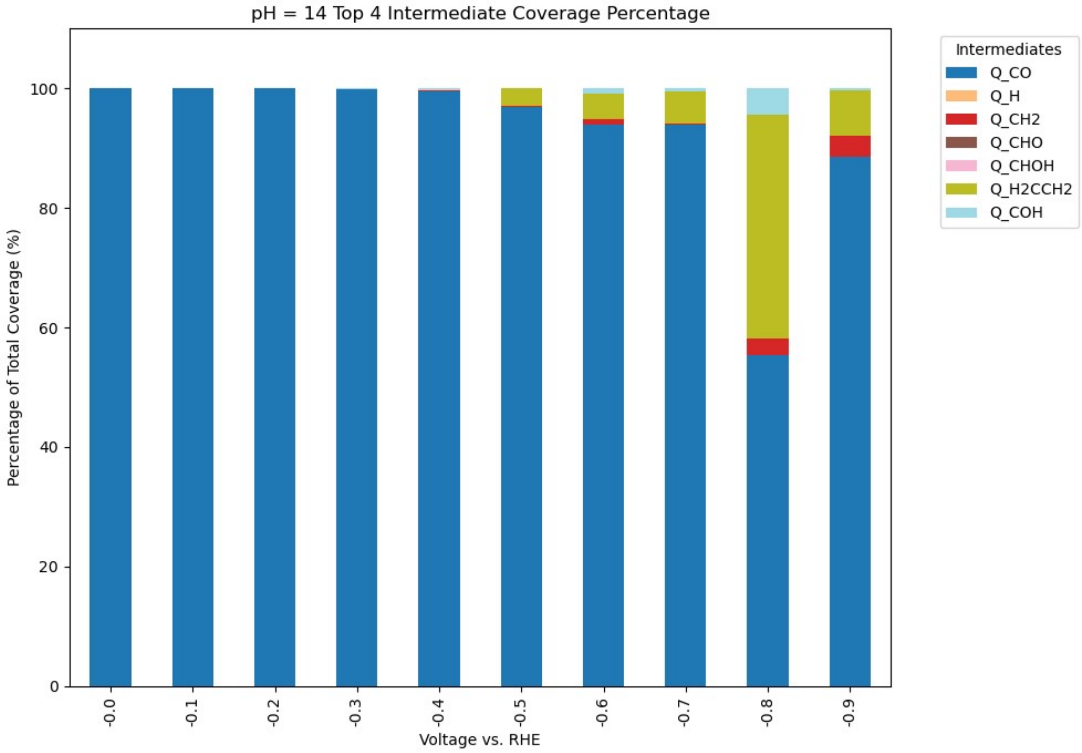

Fig. S30 The coverage ratio of different intermediates of different voltage on Cu(111) surface at pH 14.

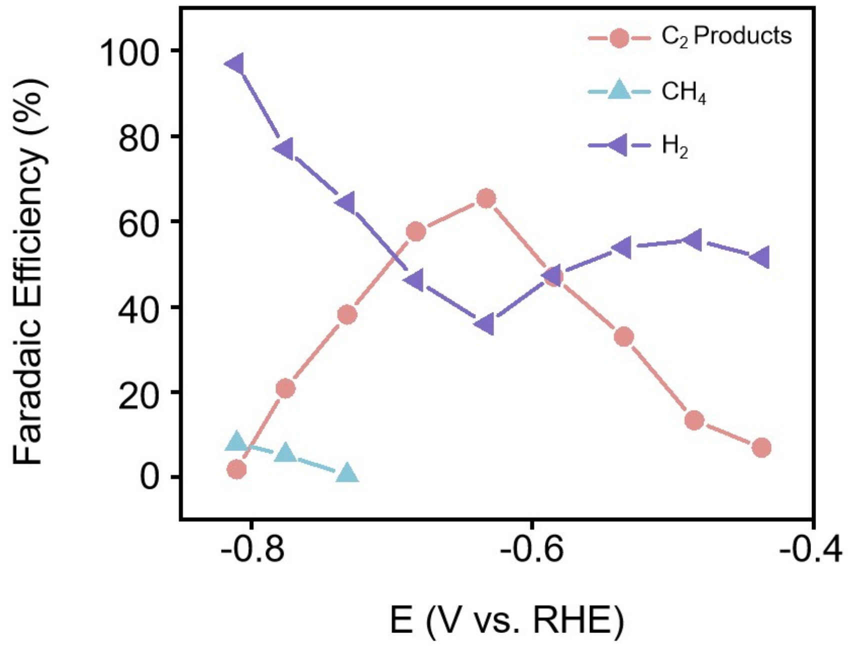

Fig. S31 Faradaic efficiencies for COR products and hydrogen as a function of applied potential. The data was obtained from previous experimental work.[5]

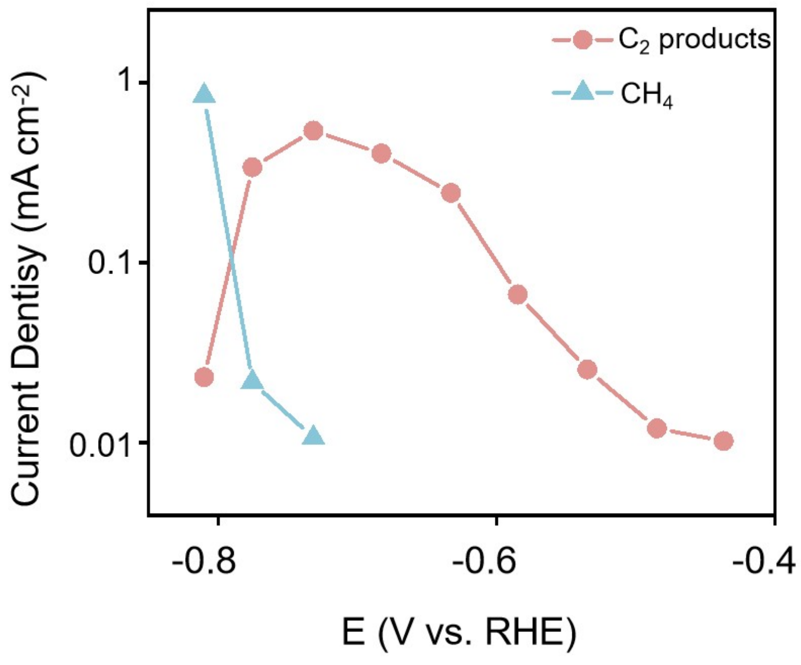

Fig. S32 Partial current densities for COR products and hydrogen as a function of applied potential. The data was obtained from previous experimental work.[5]

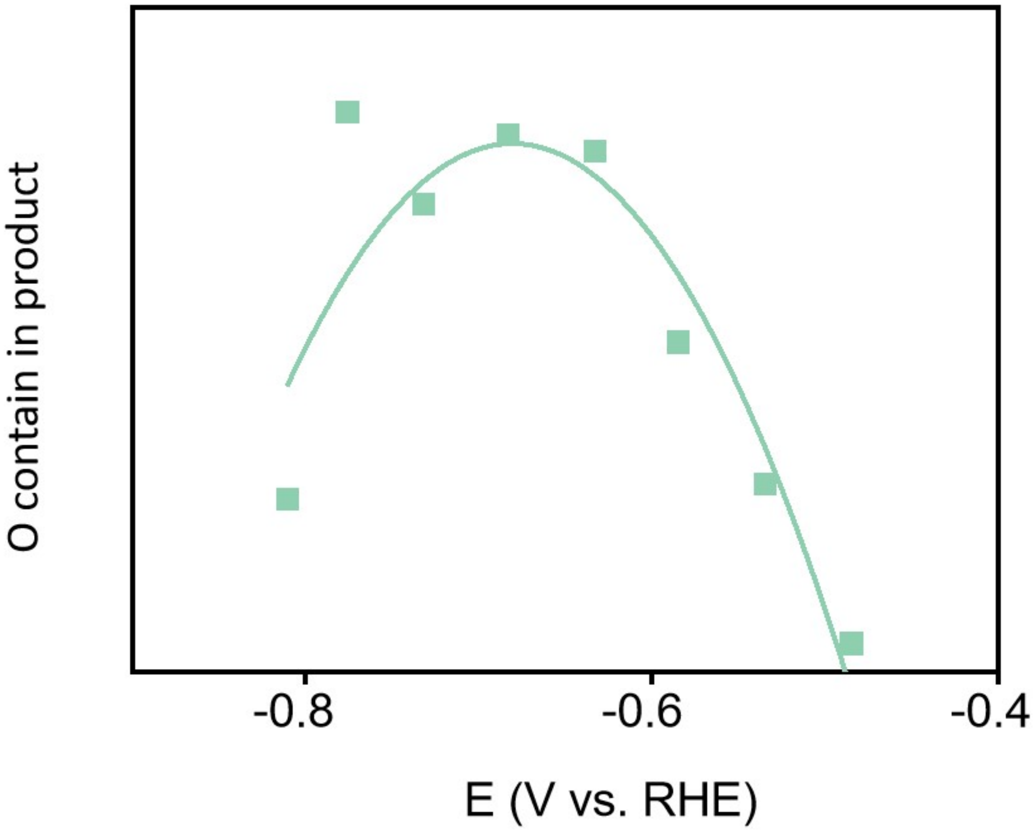

Fig. S33 The oxygen content in the products changes with the voltage. The data was obtained from previous experimental work.[5] The calculation method involves multiplying the current by the number of oxygen atoms in the product.

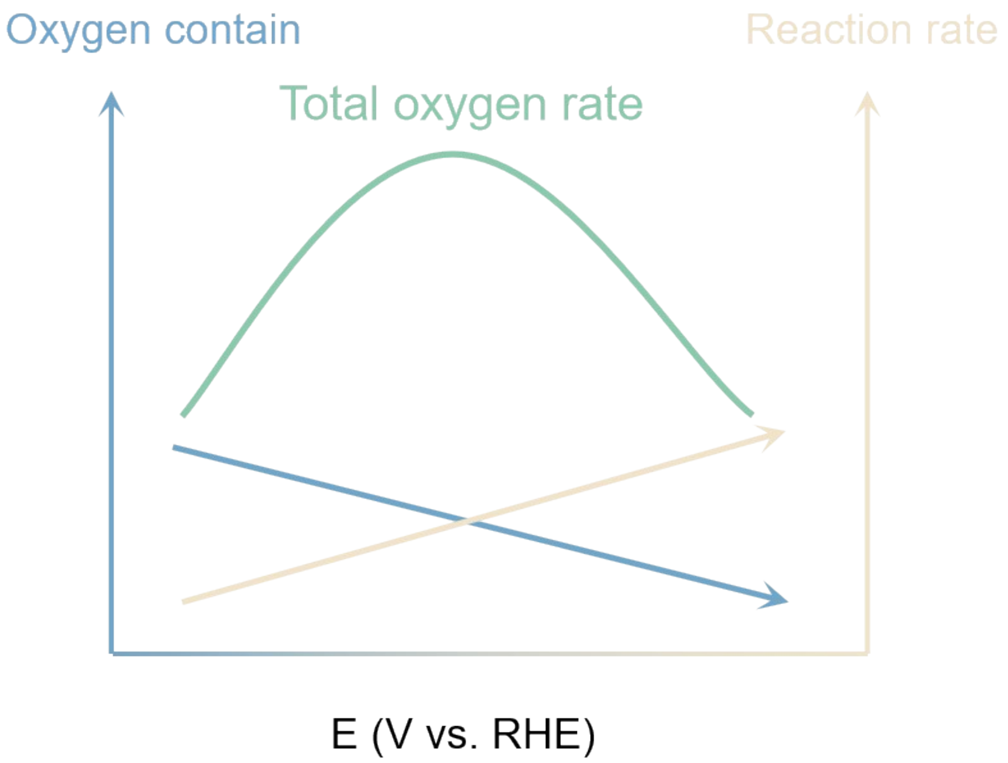

Fig. S34 Volcano curves of oxygen contain intermediate production rates.

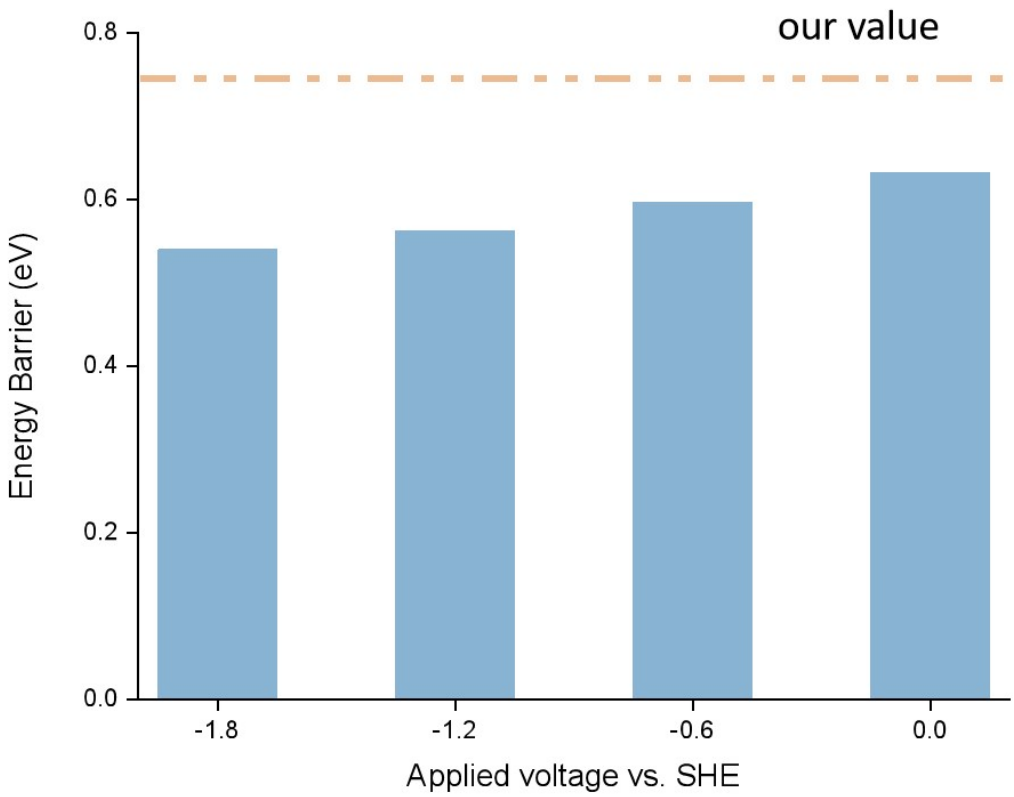

Fig. S35 The *OC-CO coupling barrier under different voltage vs. SHE calculated by

the JDFTx.

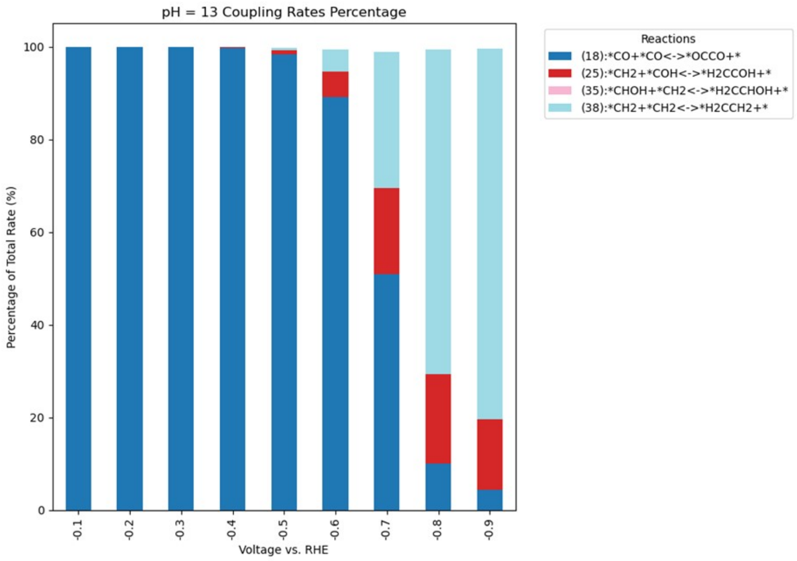

Fig. S36 The top 3 coupling mechanisms at different voltages vs. RHE (pH 13) were identified by setting the activation energy barrier of *OC-CO as 0.4 eV while keeping all other energy barriers unchanged."

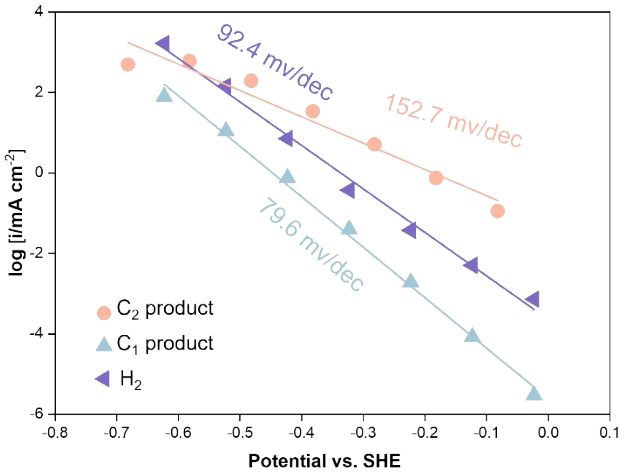

Fig. S37. The Tafel slope of the C_2/C_1/HER partial current densities obtained by the microkinetic modelling.

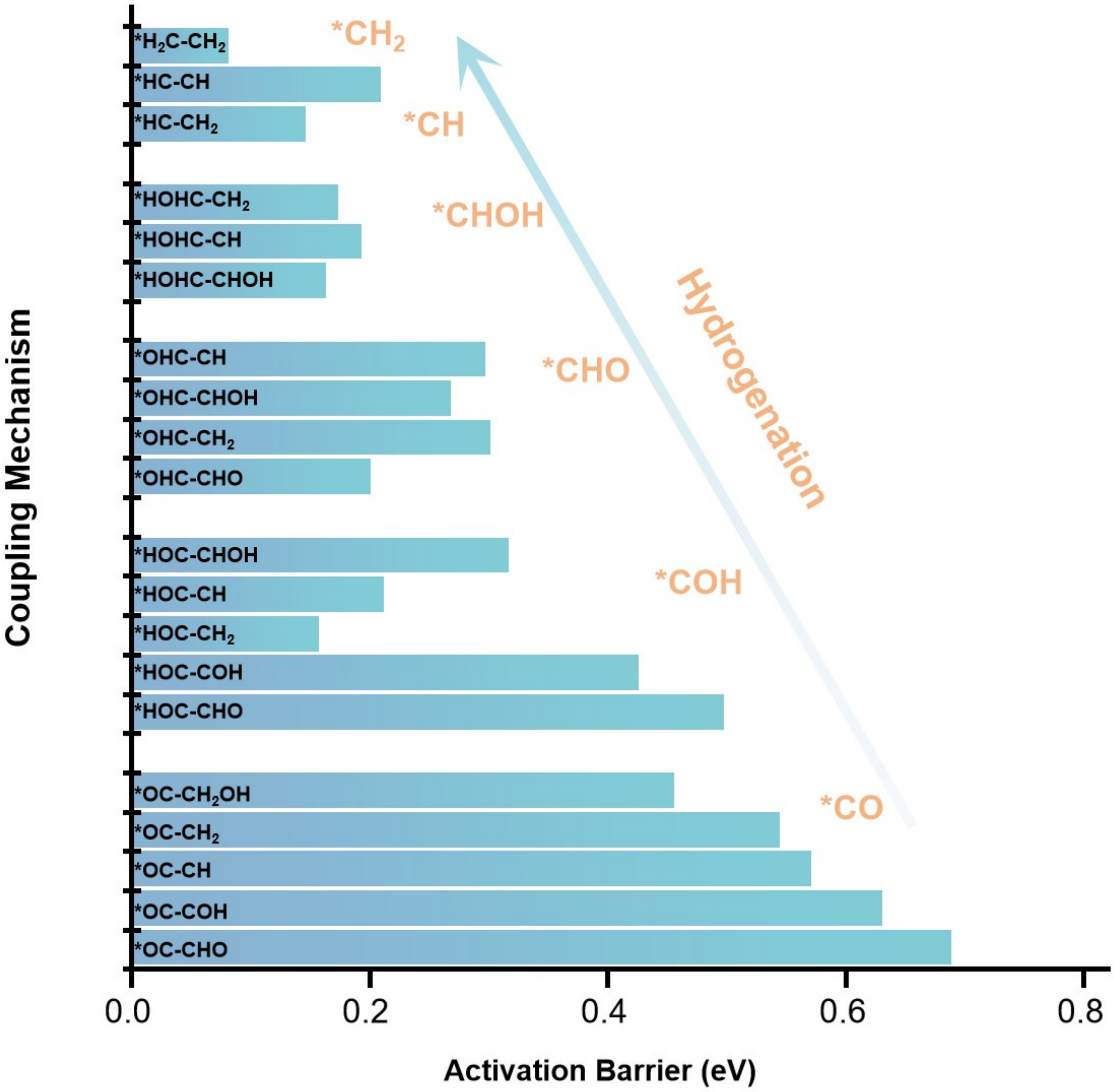

Fig. S38 The activation barrier of the 20 C-C coupling mechanism on Cu(111) surface, except *OC-CO coupling, under the implicit solvation model by VASPSOL.

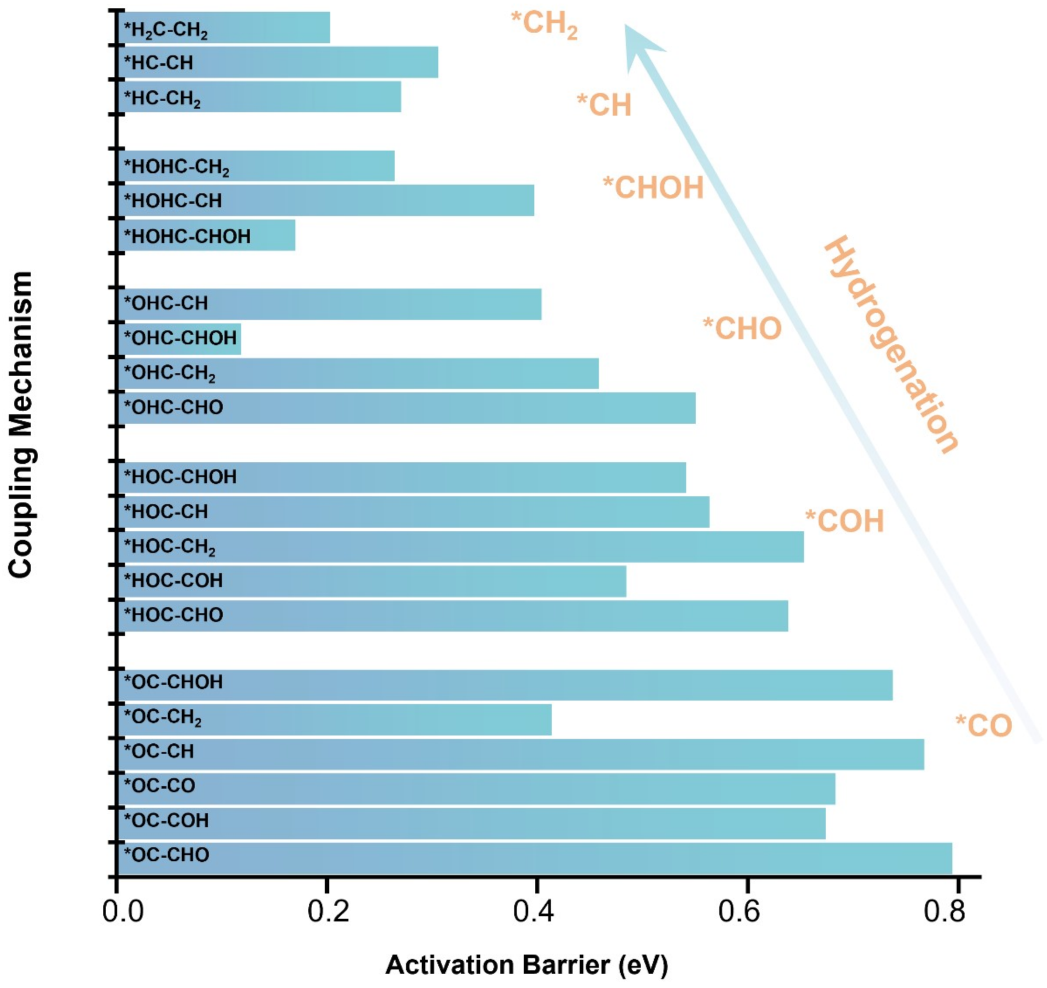

Fig. S39 The activation barrier of the 21 C-C coupling mechanism on Cu(100) surface under explicit solvation model.

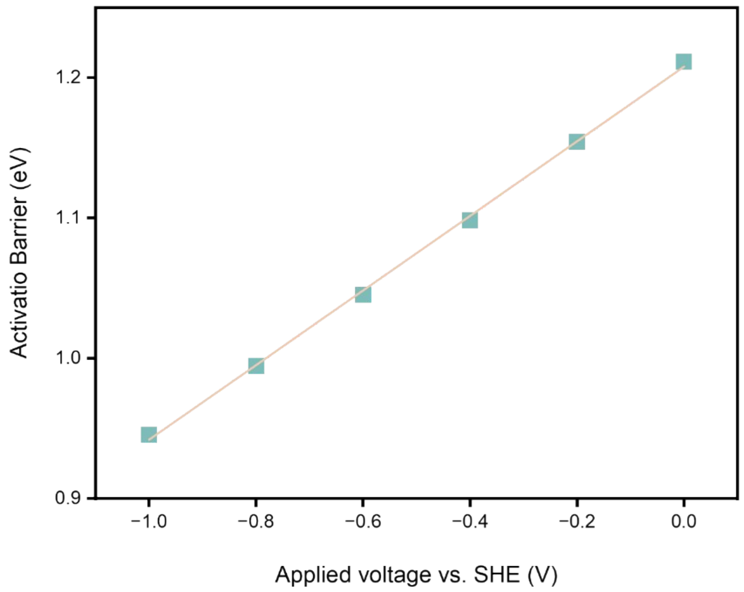

Fig. S40 The change of the activation of hydrogenation towards *COH under different potential vs. SHE.

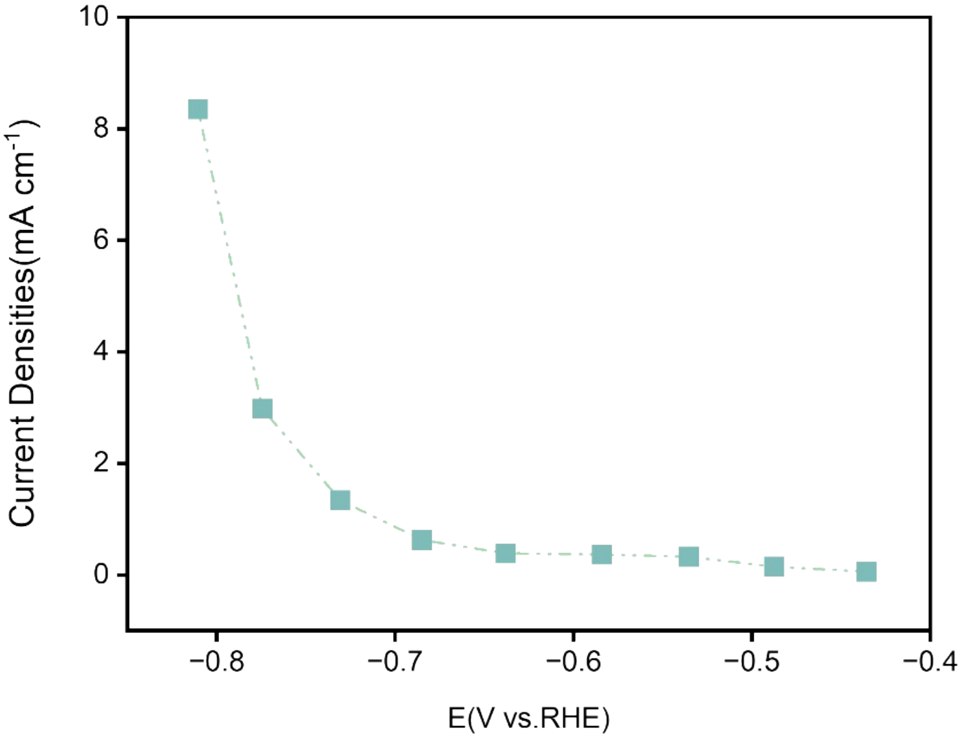

Fig. S41. The current density of H_2 under different potential vs. RHE.

Table S1. All related reactions.

(1): CO+*	↔	*CO
(2): *CO+H_2O(c)	↔	*COH+OH(c)
(3): *CO+H_2O(c)	↔	*CHO+OH(c)
(4): *COH+H_2O(c)	↔	*CHOH+OH(c)
(5): *CHO+H_2O(c)	↔	*CHOH+OH(c)
(6): *CHOH	↔	*CH+OH(c)
(7): *CH+H_2O(c)	↔	*CH_2+OH(c)
(8): *CH_2+H_2O(c)	↔	*CH_3+OH(c)
(9): *CH_3+H_2O(c)	↔	*CH_4(g)+OH(c)+*
(10): *CO+*H	↔	*COH+*
(11): *CO+*H	↔	*CHO +*
(12): *COH+*H	↔	*CHOH+*
(13): *CHO+*H	↔	*CHOH +*
(14): *CHOH+*H	↔	*CH +H_2O+*
(15): *CH+*H	↔	*CH_2+*
(16): *CH_2+*H	↔	*CH_3+*
(17): *CH_3+*H	↔	CH_4(g)+2*
(18): *CO + *CO	↔	*OCCO+*
(19): *COH + *CO	↔	*HOCCO+*
(20): *CHO + *CO	↔	*OHCCO+*

(21): *CH + *CO	↔	*HCCO+*
(22): $^*CH_2$ + *CO	↔	$^*H_2CCO$+*
(23): *COH + *CHO	↔	*OHCCOH+*
(24): *COH + *COH	↔	*HOCCOH+*
(25): $^*CH_2$ + *COH	↔	$^*H_2CCOH$+*
(26): *CHO + *CHO	↔	*OHCCHO+*
(27): * CH + *CHO	↔	*HCCHO+*
(28): * CH_2 + *CHO	↔	$^*H_2CCHO$+*
(29): * CHO + *CHOH	↔	*OHCCHOH+*
(30): * CHOH + *CHOH	↔	*HOHCCHOH+*
(31): * CHOH + *CO	↔	*OCCHOH+*
(32): *CHOH + *COH	↔	*HOCCHOH+*
(33): *CH + *COH	↔	*HCCOH+*
(34): *CHOH + *CH	↔	*HCCHOH+*
(35): *CHOH + $^*CH_2$	↔	$^*H_2CCHOH$+*
(36): *CH + $^*CH_2$	↔	$^*H_2CCH$+*
(37): *CH + *CH	↔	*HCCH+*
(38): $^*CH_2$ + $^*CH_2$	↔	$^*H_2CCH_2$+*
(39): *OCCO+6H_2O(c)	↔	$^*H_2CCH_2$+8OH(c)
(40): *HOCCO+5H_2O(c)	↔	$^*H_2CCH_2$+7OH(c)
(41): *OHCCO+5H_2O(c)	↔	$^*H_2CCH_2$+7OH(c)
(42): *HCCO+4H_2O(c)	↔	$^*H_2CCH_2$+5OH(c)

(43): *$H_2CCO+3H_2O(c)$	↔	*$H_2CCH_2+4OH(c)$
(44): *$OHCCOH+4H_2O(c)$	↔	*$H_2CCH_2+6OH(c)$
(45): *$HOCCOH+4H_2O(c)$	↔	*$H_2CCH_2+6OH(c)$
(46): *$H_2CCOH+2H_2O(c)$	↔	*$H_2CCH_2+3OH(c)$
(47): *$OHCCHO+4H_2O(c)$	↔	*$H_2CCH_2+6OH(c)$
(48): *$HCCHO+3H_2O(c)$	↔	*$H_2CCH_2+4OH(c)$
(49): *$H_2CCHO+2H_2O(c)$	↔	*$H_2CCH_2+3OH(c)$
(50): *$OHCCHOH+H_2O(c)$	↔	*$H_2CCH_2+3OH(c)$
(51): *$HOHCCHOH+2H_2O(c)$	↔	*$H_2CCH_2+4OH(c)$
(52): *$OCCHOH+4H_2O(c)$	↔	*$H_2CCH_2+6OH(c)$
(53): *$HCCOH+3H_2O(c)$	↔	*$H_2CCH_2+4OH(c)$
(54): *$HOCCHOH+3H_2O(c)$	↔	*$H_2CCH_2+5OH(c)$
(55): *$HCCOH+3H_2O(c)$	↔	*$H_2CCH_2+4OH(c)$
(56): *$HCCHOH+2H_2O(c)$	↔	*$H_2CCH_2+3OH(c)$
(57): *$H_2CCHOH+H_2O(c)$	↔	*$H_2CCH_2+2OH(c)$
(58): *$H_2CCH+H_2O(c)$	↔	* $H_2CCH_2+OH(c)$
(59): *$HCCH+2H_2O(c)$	↔	*$H_2CCH_2+2OH(c)$
(60): *H_2CCH_2	↔	$C_2H_4(g)$+*
(61): $H_2O(c)$ +*	↔	*H+ OH(c)
(62): $H_2O(c)$ +*H	↔	$H_2(g)$+ *+OH(c)
(63): *H +*H	↔	$H_2(g)$+ 2*

3. Supplementary References

1 R. Sundararaman, W. A. Goddard, 3rd and T. A. Arias, Grand canonical electronic density-functional theory: Algorithms and applications to electrochemistry, *J. Chem. Phys.*, 2017, **146**, 114104.

2 G. Kastlunger, L. Wang, N. Govindarajan, H. H. Heenen, S. Ringe, T. Jaramillo, C. Hahn and K. Chan, Using pH Dependence to Understand Mechanisms in Electrochemical CO Reduction, *ACS Catal.*, 2022, **12**, 4344-4357.

3 R. B. Sandberg, J. H. Montoya, K. Chan and J. K. Nørskov, CO-CO coupling on Cu facets: Coverage, strain and field effects, *Surf. Sci.*, 2016, **654**, 56-62.

4 J. Hou, X. Chang, J. Li, B. Xu and Q. Lu, Correlating CO Coverage and CO Electroreduction on Cu via High-Pressure in Situ Spectroscopic and Reactivity Investigations, *J. Am. Chem. Soc.*, 2022, **144**, 22202-22211.

5 L. Wang, S. A. Nitopi, E. Bertheussen, M. Orazov, C. G. Morales-Guio, X. Liu, D. C. Higgins, K. Chan, J. K. Nørskov, C. Hahn and T. F. Jaramillo, Electrochemical Carbon Monoxide Reduction on Polycrystalline Copper: Effects of Potential, Pressure, and pH on Selectivity toward Multicarbon and Oxygenated Products, *ACS Catal.*, 2018, **8**, 7445-7454.
